# Safety and immunogenicity of the invasive non-typhoidal Salmonella (iNTS)-GMMA vaccine: a first-in-human, randomised, dose escalation trial

**DOI:** 10.1016/j.ebiom.2025.105903

**Published:** 2025-09-03

**Authors:** Brama Hanumunthadu, Tesfaye Demissie, Melanie Greenland, Peter Skidmore, Kiarash Tanha, Tim Crocker-Buque, Nelly Owino, Antonella Silvia Sciré, Chiara Crispino, Daniele De Simone, Marta Benincasa, Maria Grazia Aruta, Omar Rossi, Anna Maria Colucci, Francesco Berlanda Scorza, Ashwani Kumar Arora, Xinxue Liu, Elizabeth A. Clutterbuck, Leila Godfrey, Rocio Canals, Maheshi N. Ramasamy, Vacc-iNTS Consortium, Francis Agyapong, Gianluca Breghi, Annalisa Ciabattini, John A. Crump, Melita A. Gordon, Liselotte Hardy, Samuel Kariuki, Stefano Malvolti, Carsten Mantel, Christian S. Marchello, Florian Marks, Donata Medaglini, Tonney S. Nyirenda, Mercy Ngetich, Ellis Owusu-Dabo, Francesco Santoro, J. Anthony G. Scott, Bassiahi Abdramane Soura, Tiziana Spadafina, Bieke Tack

**Affiliations:** a Oxford Vaccine Group, Department of Paediatrics, University of Oxford, Oxford, United Kingdom; b GSK Vaccines Institute for Global Health, Siena, Italy; c Oxford University Hospitals NHS Foundation Trust, Oxford, United Kingdom; d NIHR Oxford Biomedical Research Centre, Oxford, United Kingdom

**Keywords:** iNTS, Salmonella vaccine

## Abstract

**Background:**

Invasive non-typhoid *Salmonella* (iNTS) is a leading cause of morbidity and mortality in sub-Saharan Africa. We assess the safety and immunogenicity of an outer membrane vesicle vaccine (iNTS-GMMA) derived from the two most common serovars, *S.* Enteritidis (SEn) and *S.* Typhimurium (STm).

**Methods:**

This single centre, randomised within cohort, placebo-controlled dose escalation single-blind with blinded assessment trial included healthy people aged 18–55 according protocol eligibility criteria. A sentinel cohort (Group 1) was randomised 1:1 to a lower dose (10.6 μg total O-antigen [OAg]) or placebo, a subsequent cohort was randomised 1:1 to the full dose (40 μg total OAg) or placebo (Group 2), and the last cohort was randomised 2:1 (Group 3) to the full dose (40 μg total OAg) or placebo at CCVTM, University of Oxford. Participants received three intra-muscular administrations at 0, 2 and 6 months. EudraCT Number 2020-000510-14.

**Findings:**

Between May and November 2022, 7 participants were assigned to Group 1, 6 to Group 2 and 18 to Group 3. 26/31 completed follow-up at 12 months. No SAEs or SUSARs were reported. The most common adverse events (AE) were injection site reactions. All participants (19/19, 100%) in the iNTS-GMMA groups reported at least one solicited AEs, which were mostly mild to moderate in severity. 28 days following vaccination, unsolicited AEs at least possibly related to iNTS-GMMA were predominantly mild (6, 50%) and (4, 33.3%) moderate. An increase from baseline in serovar-specific OAg IgG levels peaked at day 28 following full dose (SEn: GMC 865.4 [95% CI 404.9, 1849.6]; STm: 833.2 [401.8, 1727.9]) compared with placebo (SEn: 73.7 [22.4, 242.3]; STm: 41.1 [17.6, 95.5]). Serum bactericidal antibody (SBA) peaked at day 28 following first vaccination (SEn: 38,722.7 [14,209, 105,528.1]; STm: 29,989 [18,528.6, 48,537.9]) compared with placebo (SEn: 9976 [4261.1, 23,355.5]; STm: 6694.3 [2742, 16,343.6]).

**Interpretation:**

The iNTS-GMMA vaccine was immunogenic and did not show safety concerns precluding further development, supporting progression to further phase I and II clinical trials.

**Funding:**

10.13039/501100007601EU Framework Programme for Research and Innovation grant, Horizon 2020 (grant agreement number 815439).

**Research in context**

**Evidence before this study**

We searched for *Salmonella* clinical trials on Clinicaltrials.gov and PubMed using the search terms “iNTS”, “Non-Typhoid *Salmonella*”, and “Vaccine”, followed by “OMV” and “Vaccine” up to August 2024. No results have been published from iNTS vaccine trials in humans. Prior GMMA-based vaccines include the *Shigella sonnei* GMMA (1790GAHB) vaccine. This vaccine has proven safe and immunogenic in phase I and II trials [NCT02017899, NCT02034500, NCT02676895, NCT03089879] but was not efficacious in a controlled human infection study [NCT03527173]. All studies demonstrated increased LPS IgG above baseline in those vaccinated. A second-generation *Shigella* 4-valent GMMA-based vaccine (altSonflex1-2-3) has recently completed a phase I/II clinical trial (NCT05073003). Although less well established than polysaccharide and conjugate equivalents, OMV vaccines have proven safe and immunogenic, with at least one licenced vaccine in common use [meningococcal B disease vaccine (*Bexsero*)].

**Added value of this study**

With limited available data on the human response to Non-Typhoidal *Salmonella* vaccines, this phase I clinical trial of the iNTS-GMMA vaccine demonstrates an acceptable safety profile and elevated anti-SEn and STm OAg IgG and serum bactericidal response to both *Salmonella* Enteritidis (SEn) and *Salmonella* Typhimurium (STm). In particular, this study demonstrates a human bactericidal immune response to the STm ST313 sequence type prevalent in sub-Saharan Africa, known to cause invasive disease.

**Implications of all the available evidence**

While no vaccines are currently licenced against iNTS, several early phase vaccine trials are in progress (iNTS-GMMA bivalent vaccine [NCT06213506], iNTS-TCV trivalent vaccine [NCT05480800], TSCV trivalent vaccine [NCT05784701]), and two completed (TSCV trivalent phase 1 vaccine [NCT05525546, NCT03981952]). Continued advancement is required to succeed in developing a vaccine against this neglected disease.

## Introduction

Non-typhoid *Salmonella* spp. (NTS) are Gram-negative bacteria commonly infecting domestic livestock and transmitted to humans by the orofaecal route. While most infections result in self-limiting gastrointestinal symptoms, invasion across the enteric mucosa results in bacteraemia and invasive non-typhoidal *Salmonella* disease (iNTS). Globally, 535,000 cases of iNTS occur annually, with 77,500 deaths and 4.26 million Disability-adjusted life years (DALYs) lost.^1^ The highest burden of disease occurs in sub-Saharan Africa, where iNTS accounts for more deaths and DALYs lost than *S.* Typhi,^1^ with young children under 2, people living with HIV (PLWH) and recent malaria most at risk.^2^ *Salmonella enterica* serovar Typhimurium (STm) is the leading cause of iNTS and, together with *S. enterica* serovar Enteritidis (SEn), accounts for 75% of invasive African isolates.^3^ African iNTS strains are genetically distinct from those causing gastroenteritis, with evidence of genome degradation and human host adaptation facilitating an invasive phenotype.^4^^,^^5^ Whole genome sequencing of invasive isolates confirms the emergence of a dominant STm clade (ST313-L2) over the last four decades in Africa, likely in parallel with the HIV pandemic.^6^^,^^7^ ST313-L2 isolates additionally harbour plasmid and chromosomally encoded markers for fluoroquinolone and macrolide resistance and extended-spectrum beta-lactamase activity (ESBL),^6^ and iNTS is thus designated a WHO high-priority bacterial pathogen.^8^ iNTS typically presents non-specifically with fever the most common symptom,^9^ which makes reliable diagnosis challenging in low-resource settings without access to blood culture facilities and where alternative causes of undifferentiated febrile illness such as malaria are common. Given the complexities of implementing and maintaining Water Sanitation and Hygiene (WASH) interventions to reduce transmission, vaccination remains a cost-effective route to tackling the iNTS disease burden. However, unlike other enteric pathogens such as *S.* Typhi, where vaccination programmes are being implemented globally, no licenced vaccine currently exists for iNTS.

SEn and STm are unencapsulated, unlike *S.* Typhi where vaccines are based on capsular Vi polysaccharide. However, the O antigen (OAg) component of outer membrane lipopolysaccharide (LPS) is considered an important immunogen and hence a vaccine target for iNTS pathogens.^10^ The iNTS-GMMA vaccine is based on the Generalised Module of Membrane Antigen (GMMA) technology developed by GSK Vaccines Institute for Global Health (GVGH). GMMA particles are outer membrane vesicles (OMV)–spherical structures naturally released by Gram-negative bacteria with postulated roles including immune decoy, waste disposal and quorum sensing.^11^ GMMA particles present bacterial surface proteins and LPS (including OAg) in their natural steric conformation.^12^ The bivalent iNTS-GMMA vaccine consists of a 1:1 mix of SEn and STm-derived GMMA particles and elicits OAg binding and bactericidal antibodies in pre-clinical studies.^13^ Mice receiving a single dose of a prototype STm-GMMA vaccine had lower bacterial loads following virulent challenge than unimmunised mice,^14^ supporting progression to clinical development. This study aims to assess the safety and immunogenicity of the bivalent iNTS-GMMA vaccine in humans.

## Methods

### Study design and participants

This first-in-human single centre, randomised within cohort, placebo-controlled dose escalation single-blind with blind assessment phase 1 trial was conducted at the Centre for Clinical Vaccinology and Tropical Medicine (Oxford, UK).^15^ Participants were recruited through print and social media advertisements. Healthy adults aged 18–55 with negative pre-vaccination tests for HIV antibodies, hepatitis B surface antigen, and hepatitis C antibodies were eligible to participate. A negative urinary pregnancy test was required at screening and immediately before enrolment for women of childbearing potential. Details of the full eligibility criteria are described in the trial protocol.^15^

### Randomisation and masking

Volunteers were sequentially enrolled into three groups by blinded study staff. In the sentinel safety Group 1, participants were randomised 1:1 to receive the lower dose iNTS-GMMA vaccine or placebo. In Group 2, participants were randomised 1:1 to receive the full dose iNTS-GMMA vaccine or placebo. In Group 3, participants were randomised 2:1 to receive the full dose iNTS-GMMA vaccine or placebo. Randomisation was achieved using a list generated from STATA version 16.0 (StataCorp LLC, Texas, USA)^16^ and the REDCap (Research Electronic Data Capture)^17^^,^^18^ randomisation module hosted at the University of Oxford, by study statisticians, data managers and staff administering vaccines. Participants, clinical investigators, and the laboratory team remained masked to vaccine allocation for the duration of the study. However, trial staff administering the vaccine (who played no further part in trial procedures including visits and outcome assessment) and study statisticians were unmasked. The unblinding of participants and the remaining study team occurred after the last participant's last visit.

### Vaccines and placebo

The iNTS-GMMA vaccine components and placebo were manufactured at an outsourced Contract Manufacturing Organization (CMO) and released for use in the clinical trial by GSK. Both wild-type NTS (SEn and STm) strains were genetically modified to increase the production of GMMA particles via a *tolR* mutation and reduce the reactogenicity of the lipid A component of LPS via mutations in *msbB* and *pagP*.^19^ The combination of both SEn and STm GMMA form the active components of the bivalent iNTS-GMMA vaccine. The final bivalent vaccine was mixed immediately prior to administration and consisted of 1:1 ratio of SEn and STm GMMA particles formulated on Alhydrogel (0.35 mg Al^3+^/0.5 mL dose). Depending on group allocation and randomisation, participants received the lower dose vaccine (5.3 μg OAg SEn GMMA + 5.3 μg OAg STm GMMA), the full dose (20 μg OAg SEn GMMA + 20 μg OAg STm GMMA) or placebo (Alhydrogel 0.35 mg Al^3+^/0.5 mL dose alone without the active GMMA components). Vaccine doses were chosen based on pre-clinical mice immunogenicity response.^13^^,^^20^

### Procedures

Each participant received a single intramuscular injection of lower dose iNTS-GMMA vaccine, full dose-GMMA vaccine or placebo at months 0, 2, and 6 (3 administrations each). An interim safety review was performed before dose escalation to the full dose groups, and before proceeding with Group 3 vaccinations and at 2 additional timepoints in the study.^15^ The vaccine schedule was chosen to maximise immunogenicity in children under 5 years (predominant users of an iNTS vaccine) and coincide with immunisation schedules in sub-Saharan Africa.

Participants were observed for a minimum of 60 min after vaccination. Solicited (i.e., expected and defined in the protocol) adverse events (AEs) included local site reactions such as injection site pain and systemic symptoms such as fever. Unsolicited AEs were all other events not defined as solicited. Participants were instructed to complete an electronic diary to record solicited local and systemic adverse reactions for 7 days after each vaccine administration. Solicited local adverse events included injection-site pain, tenderness, swelling, redness, induration (hardness), and solicited systemic adverse events included malaise, muscle ache, joint pain, fatigue, nausea and/or vomiting, diarrhoea, abdominal pain, anorexia (loss of appetite), headache, chills, rash, feverishness (i.e., a self-reported feeling of having a fever), and objective fever (defined as an oral temperature of 37.5 °C or higher). All participants were given an emergency 24-h telephone number to contact an on-call study physician as required. Serious adverse events were recorded throughout the follow-up period of 1 year. Safety blood tests (full blood count, urea and electrolytes, liver function tests, CRP) were collected at baseline, day 7 and day 28 following each vaccination. All adverse events were graded in severity and causality based on definitions detailed in the study protocol.^15^ Blood samples collected at day 0 (first vaccination), 7, 28, 56 (second vaccination), 63, 84, 168 (third vaccination), 175, 196, and 350 were used to test immunogenicity. Procedures in the event of a SARS-CoV 2 infection and COVID-19 vaccination were included in the study protocol.^15^

### Anti-SEn and STm OAg IgG specific ELISA

Anti-*Salmonella* OAg serum IgG was measured by standardised ELISA using SEn and STm OAg as coating antigens (at final concentrations of 15 μg/mL or 5 μg/mL, respectively) and 1:5000 dilution of goat anti-human IgG-alkaline phosphatase secondary antibody (Sigma–Aldrich Cat# A3187, RRID: AB_258054) using methodology previously described.^21^ The assays used were fully characterised in terms of standard curve accuracy, linearity, specificity, precision, and limits of quantification (LLoQ). ELISA units were expressed relative to a five-parameter human antigen-specific antibody standard serum curve composed of 10 standard points and 2 blank wells (run in duplicate on each plate). One ELISA unit is defined as the reciprocal of the dilution of the standard serum that gives an absorbance value [optical density (OD) measured at 405 nm minus the OD measured at 490] equal to 1 in the assay. The anti-SEn and STm standard serum used was generated by pooling high responder sera collected as part of the study 28 days post-second vaccination (blinded) prior to analysis of clinical samples and was calibrated to the primary standard^21^ in order to meet the definition of 1 EU as the reciprocal of the dilution giving an OD equal to 1. LLoQ was 15 EU/mL for both assays.

### SEn and STm specific SBA

Individual serum samples were heat inactivated (56 °C for 30 min) and tested against *S.* Typhimurium D23580 and *S.* Enteritidis CMCC4314 in SBA assay based on luminescent readout as previously described^22^ in the presence of 50% baby rabbit complement (Cedarlane, Cat# CL3441-S100-R). The assay results were expressed as the IC50, the reciprocal serum dilution that resulted in a 50% reduction of luminescence and thus corresponding to 50% growth inhibition of the bacteria present in the assay. GraphPad Prism 10 software was used for curve fitting and IC50 determination. Both assays used were fully characterised in terms of linearity, specificity, precision, and lower limits of quantification which were equal to 51.3 and 42.3 for STm and SEn, respectively.

### Outcome

The study's primary outcome was the safety and tolerability of the iNTS-GMMA vaccine as measured by the occurrence of adverse events, including solicited and unsolicited AEs, SAEs, and SUSARs occurring throughout the study. The secondary outcome was the vaccine's immunogenicity as determined by antibody concentration against serovar-specific O antigen using the ELISA method.

Exploratory outcomes of the study included functional antibody assessment by SEn and STm serovar-specific serum bactericidal antibody (SBA) titre. A full list of exploratory endpoints is listed in the study protocol.^15^

### Statistics

The primary outcome analysis included all participants with available data. The maximum severity for each solicited systemic and local AE across the seven days after vaccination were derived for each participant and summarised by study group. Frequencies and percentages of adverse events were reported by study arm.

Immunogenicity secondary and exploratory outcome analyses included all participants who received at least one dose of vaccine in the study. Geometric mean concentrations (GMCs) and 95% confidence intervals were reported for day 0, 7, 28, 56, 63, 84, 168, 175, 196, and 350 for the IgG ELISA and day 0, 28, 84, 168, 196, and 350 for the SBA.

The primary analysis was descriptive, and no formal sample size calculation was conducted for this first-in-human phase I study. Statistical analyses did not include hypothesis testing for group comparisons. No formal significance tests were conducted. Analyses were performed using R 4.1.3 and RStudio 2023.12.1+402.

### Ethics

Written informed consent was obtained from all participants, and the trial was conducted in accordance with the principles of the Declaration of Helsinki and Good Clinical Practice. This study was approved in the UK by the Medicines and Healthcare Products Regulatory Agency (CTA 21584/0455/001-0001) and the South Central–Oxford A Research Ethics Committee (reference: 22/SC/0059). The trial registered with ISCRTN (ISRCTN51750695) and EudraCT (2020-000510-14). An independent data and safety monitoring committee (DSMC) provided safety oversight. The DSMC consisted of four members including a chairperson and statistician. Further information can be found in the protocol^15^ or DSMC charter (available on request).

### Role of funders

EU Framework Programme for Research and Innovation grant, Horizon 2020 (grant agreement number 815439). The funder of the study had no role in study design, data collection, data analysis, data interpretation or writing of the report.

## Results

### Trial conduct and demographics

**Fig. 1:**
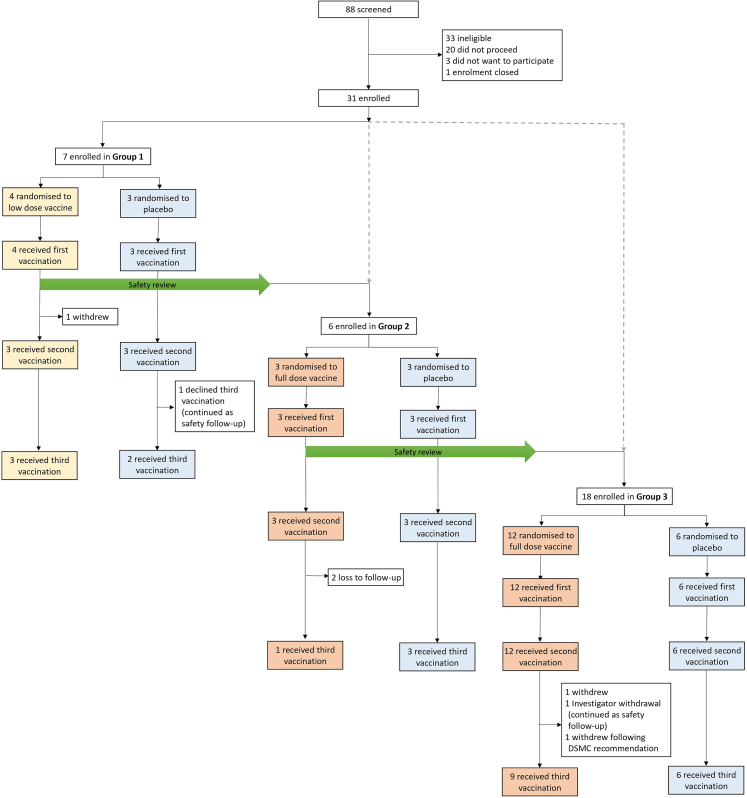
**CONSORT diagram**.

Between May and November 2022, 31 healthy adults were enrolled and subsequently randomised (Fig. 1): seven participants were sequentially enrolled into Group 1 (lower dose vaccine and placebo), six participants in Group 2 (full dose vaccine and placebo), and eighteen participants in Group 3 (full dose vaccine and placebo). An additional participant was enrolled into Group 1 due to a randomisation error, resulting in seven participants in this group rather than the planned six. Subsequent results are presented by drug allocation; 4 participants received a lower dose iNTS-GMMA vaccine, 15 participants received a full dose iNTS-GMMA vaccine, and 12 received the Alhydrogel placebo. DSMC review of safety data (SUSARs, SAEs, solicited and unsolicited AEs) at 7 days following first vaccination in group 1 allowed progression from lower dose to full dose in group 2.

In the full dose vaccine arm, 15 received the first (month 0) and second (month 2) full dose iNTS-GMMA vaccine. However, only 10 received the third vaccine at month 6 as 2 participants were lost to follow-up, 1 withdrew due to personal reasons, and 2 were withdrawn on clinical grounds (see below). In the lower dose vaccine arm, 4 received the first lower dose iNTS-GMMA vaccine, and 3 received the second and third vaccinations. However, 1 participant withdrew before the second vaccination for personal reasons. In the placebo arm, 12 received the first and second placebo administration, and 11 received the third placebo administration, with one withdrawal due to personal reasons. 26 participants completed the final follow-up visit at one year (November 2023).

No protocol-specified group-holding or individual-holding rules were met. However, two participants were withdrawn from the full dose vaccine group: one at the investigator's discretion due to persistent grade 1–2 neutropenia and another one on recommendation from the DSMC due to grade 3 local injection site reactions after both the first and second vaccinations.

The study had a skew toward male participants across each arm (67.7% male) [Table 1]. Sex was self-reported by participants. The overall median age was 27.0 (22.5–46.0) years, and 87.7% of participants identified as white.

**Table 1:** Demographics and clinical characteristics of participants enrolled by study arm.

	Placebo (N = 12)	Lower dose (N = 4)	Full dose (N = 15)	Total (N = 31)
Sex				
Male	9 (75.0%)	3 (75.0%)	9 (60.0%)	21 (67.7%)
Female	3 (25.0%)	1 (25.0%)	6 (40.0%)	10 (32.3%)
Age				
Mean (SD)	33 (12)	37 (17)	32 (12)	33 (12)
Median (Q1–Q3)	29.5 (22.0–42.8)	37.5 (23.0–51.5)	27.0 (24.0–38.5)	27.0 (22.5–46.0)
Weight (kg)				
Mean (SD)	79 (14)	90 (10)	83 (19)	83 (16)
Median (Q1–Q3)	80.7 (73.1–85.1)	94.2 (89.2–95.2)	84.0 (74.6–89.0)	84.0 (74.3–89.0)
Ethnicity				
White	12 (100.0%)	2 (50.0%)	13 (86.7%)	27 (87.1%)
Other	0 (0.0%)	2 (50.0%)	2 (13.3%)	4 (12.9%)

N = Number of Participants.

### Safety of the full dose and lower dose iNTS-GMMA vaccine

**Fig. 2:**
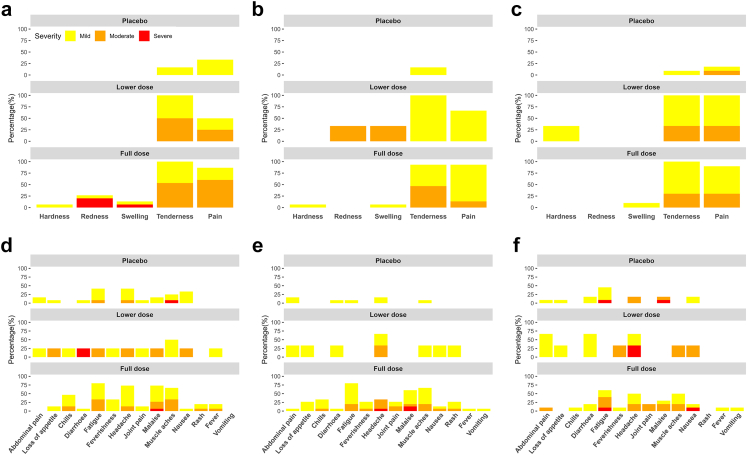
**Severity of solicited adverse reactions in days 0–7 after vaccination by study arm as self-reported in participant electronic diaries**. a: Local reactions, first vaccination; b: Local reactions, second vaccination; c: Local reactions, third vaccination; d: Systemic reactions, first vaccination; e: Systemic reactions, second vaccination; f: Systemic reactions, third vaccination. Redness, hardness, and swelling gradings are derived from the FDA Guidance for Industry: Toxicity Grading Scale for Healthy Adult and Adolescent Volunteers Enrolled in Preventive Vaccine Clinical Trials.^23^ Grading of severity: Mild (Yellow), Moderate (Orange), Severe (Red). First Vaccination: placebo n = 12, lower dose n = 4, full dose n = 15. Second Vaccination: placebo n = 12, lower dose n = 3, full dose n = 15. Third Vaccination: placebo n = 11, lower dose n = 3, full dose n = 10.

The study did not report any serious adverse events (SAEs) or severe unexpected serious adverse reactions (SUSARs). The most common solicited AEs were local reactions, with all vaccine recipients reporting mild to moderate tenderness at the injection site after each vaccination (Fig. 2, Table 2; Suppl. Table S1, Suppl. Figures S1–S3). Severe local AEs were also reported by vaccine recipients, of which 3/19 (15.8%) and 1/19 (5.3%) reported redness and swelling, respectively, at the injection site following the first vaccination. All injection site reactions, including grade 3 AEs, were transient and self-resolved within 7 days.

**Table 2:** Summary of unsolicited adverse events by study arm, placebo, lower dose, and full dose.

Characteristic	Placebo N = 30		Lower dose N = 5		Full dose N = 27		Overall N = 62	
	n (%)	95% CI	n (%)	95% CI	n (%)	95% CI	n (%)	95% CI
Number of unique participants	10 (33%)	18%, 53%	2 (40%)	7.3%, 83%	13 (48%)	29%, 68%	10 (33%)	18%, 53%
SAE	0 (0%)	–	0 (0%)	–	0 (0%)	–	0 (0%)	–
Severity								
Mild	21 (70%)	50%, 85%	3 (60%)	17%, 93%	17 (63%)	42%, 80%	41 (66%)	53%, 77%
Moderate	7 (23%)	11%, 43%	1 (20%)	1.1%, 70%	7 (26%)	12%, 47%	15 (24%)	15%, 37%
Severe	2 (6.7%)	1.2%, 24%	1 (20%)	1.1%, 70%	3 (11%)	2.9%, 30%	6 (9.7%)	4.0%, 21%
Causality								
No relationship	26 (87%)	68%, 96%	4 (80%)	30%, 99%	20 (74%)	53%, 88%	50 (81%)	68%, 89%
Possible	3 (10%)	2.6%, 28%	1 (20%)	1.1%, 70%	3 (11%)	2.9%, 30%	7 (11%)	5.0%, 22%
Probable	0 (0%)	–	0 (0%)	–	1 (3.7%)	0.19%, 21%	1 (1.6%)	0.08%, 9.8%
Definite	1 (3.3%)	0.17%, 19%	0 (0%)	–	3 (11%)	2.9%, 30%	4 (6.5%)	2.1%, 16%
Ongoing at end of study	7 (23%)	11%, 43%	0 (0%)	0	3 (11%)	2.9%, 30%	10 (16%)	8.4%, 28%
Ongoing at end of study Lost to follow up	0 (0%)	–	0 (0%)	–	2 (7.4%)	1.3%, 26%	2 (3.2%)	0.56%, 12%
Diary AE	6 (20%)	8.4%, 39%	3 (60%)	17%, 93%	10 (37%)	20%, 58%	19 (31%)	20%, 44%
Diary AE vaccination								
First	4 (67%)	24%, 94%	3 (100%)	31%, 100%	2 (20%)	3.5%, 56%	9 (47%)	25%, 71%
Second	1 (17%)	0.88%, 64%	0 (0%)	–	6 (60%)	27%, 86%	7 (37%)	17%, 61%
Third	1 (17%)	0.88%, 64%	0 (0%)	–	2 (20%)	3.5%, 56%	3 (16%)	4.2%, 40%
Not diary AE	24 (80%)	61%, 92%	2 (40%)	7.3%, 83%	17 (63%)	42%, 80%	43 (69%)	56%, 80%
AE source								
Laboratory result	9 (30%)	15%, 50%	1 (20%)	1.1%, 70%	6 (22%)	9.4%, 43%	16 (26%)	16%, 39%
Vital signs	1 (3.3%)	0.17%, 19%	0 (0%)	0.00%, 54%	1 (3.7%)	0.19%, 21%	2 (3.2%)	0.56%, 12%
Reported at visit	11 (37%)	21%, 56%	0 (0%)	0.00%, 54%	7 (26%)	12%, 47%	18 (29%)	19%, 42%
Other	4 (13%)	4.4%, 32%	1 (20%)	1.1%, 70%	3 (11%)	2.9%, 30%	8 (13%)	6.1%, 24%
COVID AE								
No	27 (90%)	72%, 97%	4 (80%)	30%, 99%	24 (89%)	70%, 97%	55 (89%)	78%, 95%
Yes	1 (3.3%)	0.17%, 19%	1 (20%)	1.1%, 70%	1 (3.7%)	0.19%, 21%	3 (4.8%)	1.3%, 14%
Unsure	2 (6.7%)	1.2%, 24%	0 (0%)	–	2 (7.4%)	1.3%, 26%	4 (6.5%)	2.1%, 16%

N = Number of Adverse Events; ‘Unsure’ = Symptoms may or may not be related to COVID-19 disease; AE = Adverse Events; SAE = Serious Adverse Events.

Fatigue, headache, malaise, and muscle aches were the most commonly reported solicited systemic AEs following full dose vaccination compared with abdominal pain, headache and muscle aches following lower dose vaccination (Fig. 2). 5 participants reported a temperature higher than 37.5 °C in the 7 days after vaccination (Suppl. Table S1, Suppl. Figures S4–S6).

Unsolicited adverse events considered to be possibly, probably, or definitely related to iNTS-GMMA were predominantly mild to moderate in nature (6/8 (75%)) and all except 2 episodes of neutropenia resolved within 28 days post-vaccination (Suppl. Tables S2 and S3).

Laboratory adverse events were similar across all groups (vaccine and placebo), with 7 reported in the vaccine recipients, of which 2 were judged related to vaccine: mild or moderate neutropenia. Of the 2 cases, 1 was transient and 1 was mild and improving by the end of the study. 1 case of neutropenia possibly related to vaccination was also reported in the placebo group (Table 2). Despite the study taking place during the COVID-19 pandemic, only 3 AEs across vaccine and placebo recipients were related to COVID-19.

### Vaccine-induced SEn and STm OAg specific IgG response

iNTS-GMMA induced a strong antibody response against SEn and STm OAg in all recipients, which persisted for 350 days post-vaccination in participants available for follow-up. Antibody responses increased rapidly after vaccination, peaking 28 days after vaccination for all groups.

**Fig. 3:**
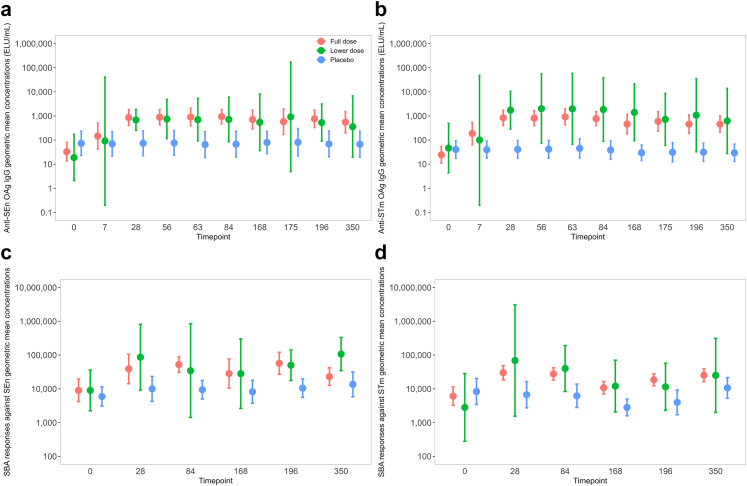
**Immune responses–geometric mean concentrations (**±**95% confidence interval) following vaccination by study arm and timepoint (day)**. a: Anti-SEn OAg IgG; b: Anti-STm OAg IgG; c: SBA responses against SEn; d: SBA responses against STm. Full dose: Day 0 (n = 15), 7 (n = 14), 28 (n = 15), 56 (n = 15), 63 (n = 13), 84 (n = 15), 168 (n = 12), 175 (n = 9), 196 (n = 12), 350 (n = 11); Lower dose: Day 0 (n = 4), 7 (n = 3), 28 (n = 4), 56 (n = 3), 63 (n = 3), 84 (n = 3), 168 (n = 3), 175 (n = 2), 196 (n = 3), 350 (n = 3); Placebo: Day 0 (n = 12), 7 (n = 12), 28 (n = 12), 56 (n = 12), 63 (n = 11), 84 (n = 12), 168 (n = 12), 175 (n = 10), 196 (n = 11), 350 (n = 11).

SEn (O:9) and STm (O:4, 5) OAg IgG antibody levels (EU/mL) measured by ELISA were similar at baseline across all treatment allocations (SEn: full dose, GMC 32.9 EU/mL, 95% CI [13.5, 80.4]; lower dose, 18.9 [2.1, 171.7]; STm: full dose, 24.4 [11, 54]; lower dose, 46.7 [4.4, 497.3]) and placebo (SEn: 73.4 [22.9, 234.9]; STm: 40.6 [17.6, 90.8])[Fig. 3, Suppl. Table S4].

Anti-SEn and STm OAg IgG GMCs following vaccination peaked at day 28 (SEn: full dose 865.4 [404.9, 1849.6], lower dose 686 [251.2, 1873.2]; STm: full dose 833.2 [401.8, 1727.9], lower dose 1734.3 [283.2, 10,623.1]) with no corresponding rise seen in placebo recipients (SEn: 73.7 [22.4, 242.3 ]; STm: 41.1 [17.6, 95.5]).

No evidence of additional boosting was seen after second and third vaccinations (Fig. 3, Suppl. Tables S4 and S5). Antibody levels against SEn and STm OAg persisted up to the final time point at day 350 (6 months following the third vaccination) in full dose recipients but the 95% CI at day 350 of low dose and placebo recipients overlapped.

### Vaccine-induced SEn and STm specific serum bactericidal antibody response

Baseline SEn and STm serum bactericidal antibody levels were similar across all treatment allocations, full dose vaccine (SEn 6073.5 [3247.8, 11,357.7]; STm 9001.8 [4181.6, 19,378.2]), lower dose vaccine (SEn 9001.7 [2244.3, 36,104.8]; STm 2812.9 [279.8, 28,276.9]) and placebo (SEn 5922.7 [3068.9, 11,430.1]; STm 8339.5 [3442.3, 20,203.8]) [Fig. 3, Suppl. Table S5].

In full and lower dose iNTS-GMMA vaccine arms, both SEn and STm bactericidal antibodies peaked at day 28 following the first vaccination (SEn: full dose SBA GMT 38,722.7 [95% CI 14,209, 105,528.1], lower dose 86,436.3 [9214.8, 81,0783.2]; STm: full dose 29,989 [18,528.6, 48,537.9], lower dose 69,153.2 [1555.5, 3,074,275.3]), placebo (SEn: 9976 [4261.1, 23,355.5]; STm: 6694.3 [2742, 16,343.6]). Both SEn and STm SBA titres tended to decline by day 350, but remained above baseline levels in vaccine recipients, full dose (SEn: 23,000.8 [12,625.5, 41,902.3]; STm: 25,333.7 [16,403.3, 39,126.1]), and lower dose (SEn: 107,338 [34,539.4, 333,574.4]; STm: 24,967.5 [1997.2, 312,123.2]).

The seroresponse rate, as defined by the percentage of participants who mounted a post-vaccination titre equal to or greater to four times baseline serum bactericidal antibody, was similar for SEn and STm at 28 days (SEn: full dose 40% [95% CI 16%, 68%], lower dose 75% [19%, 99%]; STm: full dose 60% [32%, 84%], lower dose 50% [6.8%, 93%]) and at 350 days (SEn: full dose 36% [11%, 69%], lower dose 67% [9.4%, 99%]; STm: full dose 55% [23%, 83%], lower dose 33% [0.84%, 91%]) post-vaccination [Suppl. Table S6].

## Discussion

In this study, we have demonstrated that the iNTS-GMMA vaccine given in a 3-dose regimen has a tolerable systemic safety profile in the limited participants recruited, although there was evidence of local injection site reactogenicity. The majority of adverse events reported were mild to moderate in nature and all solicited adverse events were self-limiting. Two participants were withdrawn as a precaution from further vaccination due to a local injection site inflammatory reaction and grade 2 asymptomatic neutropenia.

The profile of adverse events following the iNTS-GMMA vaccination is similar to that of another GMMA platform vaccine against *Shigella*.^24^ Systemic symptoms were not prominent, and fever was reported by a small number of participants. The meningococcal B OMV vaccine (*Bexsero*), one of the only licenced OMV-based vaccines in the UK, has a similar side effect profile, with injection site reactions seen commonly.^25^^,^^26^ However, the side effect profile varies by age, with fever more prominent in infants than adults. Further studies in children under five, the primary target population of an iNTS-GMMA vaccine, would be required to confirm the safety profile in this age group as well as a larger sample size. Neutropenia was an adverse event of special interest in the *S. sonnei* GMMA booster extension study,^27^ with some participants experiencing transient asymptomatic neutropenia.^24^ However, a further analysis using ethnicity appropriate neutrophil thresholds showed no increased risk of neutropenia with the *S. sonnei* candidate vaccine in adults compared with placebo or other licenced vaccines.^28^ Neutropenia was infrequent in this iNTS-GMMA study, however safety monitoring will continue in future planned studies.

Enrolment occurred during the later stages of the COVID-19 pandemic, but this had little impact on trial conduct and delivery. We observed few COVID-19-related AEs, including a participant who developed a SARs-CoV-2 infection within seven days of the first dose of iNTS-GMMA vaccination, which may have contributed to reported AEs.

Bacterial outer membrane LPS is a pathogen-associated molecular pattern (PAMP) recognised by cell surface pattern recognition receptors (PRR), of which toll-like receptors are among the most important.^19^ Triggering of PRR on innate effector cells, including monocytes by PAMPs, and subsequent recruitment and activation of these cells may explain the local inflammatory reactions seen at the injection sites following vaccination.^29^ In addition to the modification of the *tolR* gene to increase production of GMMAs,^30^ the GMMA technology involves modification of *msbB* and *pagP* genes in parent bacteria to alter acylation of the lipid A component of LPS,^19^ thereby reducing reactogenicity in pre-clinical models.^31^ Our data suggest that further studies needed to establish the safety profile of the vaccine in humans.

While no formal between-group or timepoint statistical calculations were performed, the iNTS-GMMA vaccine was immunogenic at both doses, with responses demonstrating good durability for up to 1 year. The onset of humoural response was rapid, with OAg-binding antibody and SBA activity rising from day 7 and peaking at 28 days after the first vaccination. The early response following vaccination and detectable levels at baseline suggest a boosted rather than primary response in most participants. The wide CI seen in the sentinel lower dose group (sample size) precludes any interpretation of the difference in immunogenicity between the two dose levels. The bivalent iNTS-GMMA vaccine uses GMMA particles from both SEn and STm serovars—we did not observe any evidence of interference between individual serovar-specific anti-OAg IgG or SBA responses in vaccine recipients.

In African infants, acquiring anti-OAg IgG and bactericidal antibodies in the first year of life correlates with protection from iNTS.^10^ While no formal humoural correlate of protection has yet been identified, it is encouraging that the iNTS-GMMA vaccine may mimic this natural acquisition of antibodies from exposure to environmental *Enterobacteriaceae*. Pre-existing IgG antibodies to SEn and STm OAg with bactericidal activity were observed at baseline, likely due to cross-reacting antibodies. This is consistent with prior seroepidemiology studies in the UK,^32^ which suggest that most adults have been exposed to *Salmonella* (*similarly seen in the Shigella GMMA vaccine studies*^24^^,^^27^^,^^28^).

While the GMMAs were produced from genetically modified SEn 618 and STm 2192 strains, the generated human antibodies displayed bactericidal activity against the African STm D2350 (ST313) strain, confirming findings of previous animal models.^20^^,^^31^

OAg is the most likely immunodominant component of GMMA particles, with serovar-specific anti-OAg IgG being likely the greatest contributor to SBA activity.^13^ However, human serum resistance has already emerged to STm ST313 in sub-Saharan Africa due to the elongation of the O-antigen component.^33^ Should resistance arise to O-antigen-based vaccines, an outer membrane vesicle vaccine may remain effective due to the presentation of multiple antigens and the potential to stimulate a polyclonal response.

*Salmonella* is an intracellular pathogen which may explain the association of disease in PLWH with impaired T cell responses^34^ and in those with impaired phagocytic function following malaria infection.^35^ While the development of antigen-specific CD3+CD4+ T cells alone are not associated with protection against iNTS,^10^ T cell help is needed for effective class switching and generation of bactericidal antibodies. We postulate that the iNTS-GMMA vaccine has the potential to elicit robust T cell-dependent immune responses similar to traditional polysaccharide-protein conjugate vaccines, which exploit the phenomenon of linked recognition, as OAg and proteins in GMMA particles will be taken up together by antigen-presenting cells. Future work will interrogate antigen specific T cell responses from vaccinees to proteins found in GMMAs and parent bacteria.

The population most at risk of iNTS-are children under five in sub-Saharan Africa.^19^ However, as is standard in early phase vaccine trials due to the lack of safety data, this study was conducted in healthy adults. We therefore used a 3 dose regime as is typical for other childhood vaccines such as the pneumococcal conjugate vaccine (PCV) which is needed to elicit robust immune responses in infants. Any future iNTS vaccine aimed at this demographic will therefore need to consider the optimum time point for incorporation into already crowded infant immunisation schedules^36^ as well as the likely immunogenicity generated from a primary rather than boosted response. The development of multivalent *Salmonella* vaccines combining iNTS antigens with *S.* Typhi or *S.* Paratyphi, may minimise needle burden and vaccine cold storage capacity in resource poor settings. However, careful consideration will need to be given of the geographical localisation and age distribution of susceptible children to each of these *Salmonella* serovars.^37^ Clinical trials of trivalent *Salmonella* vaccines are under investigation,^38^ including a study involving a trivalent vaccine incorporating a combination of the iNTS-GMMA and typhoid-conjugate vaccine.^39^

### Study limitations

Limitations of this study include the small sample size and restricted demographics of the study participants. Long-term safety and immunogenicity findings should be interpreted cautiously, given 6/31 participants did not receive all three planned vaccines. Data analysis was undertaken following a database-lock, once all reasonable efforts were made to identify and resolve missing data, including reminders to participants and observers at follow-up visits and telephone reviews. Except for these 6/31 participants, further missing data was insubstantial, and did not affect the conclusion drawn from the study. As a first-in-human trial, the safety study design necessitated the administration of a lower dose prior to full dose vaccine in a small cohort of participants, as a result observers were aware of the groups which may have received lower dose (versus placebo) or full dose (versus placebo). Therefore, an observation bias may have been introduced in the comparison of lower dose versus full dose. In addition, the lower dose vaccine sample size was too small for a conclusive comparison. However, a comparison of these two dose levels was not strictly a primary or secondary outcome of this trial and may be further investigated in further phase I/II studies. While the study was conducted in an adult European cohort, the targeted demographic will primarily be children in sub-Saharan Africa. Further studies are required in this group to confirm both safety and immunogenicity. Demographic differences between groups might include multiple host factors, including prior exposure to both *S.* Typhi, SEn and STm, which might affect immunogenicity to the iNTS-GMMA vaccine. Further research is required to better understand the importance of pre-existing immunity and cross-reactivity to other *Salmonellae* in the context of an endemic setting and in infants. Nonetheless, this study provides valuable information on the reactogenicity and immunogenicity of the clinical use of the iNTS-GMMA vaccine.

### Conclusion

The iNTS-GMMA vaccine did not show safety concerns precluding further development. Three doses of vaccine-elicited humoural immune responses against SEn and STm. The results of this study support progression to phase I and II studies in Africa, where a range of doses will be tested. Further analysis of immunological responses to vaccination is ongoing.

## Contributors

BH and MR wrote the manuscript. BH, MR, XL, ASS, CC, AMC, FBS, AKA, and RC were involved in study concept or design. BH, TD, PS, KT, MG, NO, TCB, EC, LG, DDS, MB, MGA, OR, and MR were involved in study conduct and/or data acquisition. KT, XL, and MG accessed and verified the underlying data. BH, MR, MG, KT, XL, ASS, CC, DDS, MB, MGA, OR, AMC, FBS, AKA, and RC were involved in data interpretation. All authors read and approved the manuscript.

All Vacc-iNTS consortium authors were involved with the study concept, provided scientific advice, reviewed and approved the manuscript.

## Data sharing statement

The study protocol is available in Hanumunthadu et al. (2023).^15^ Individual participant trial data is available upon request directed to the Oxford Vaccine Group (info@ovg.ac.uk); once approved, data access can be shared through a secure online platform.

## Declaration of interests

ASS, CC, DDS, MB, MGA, OR, AMC, FBS, AKA, and RC are/were employees of GSK. OR, AMC, FBS, AKA, and RC hold financial equities in GSK. FBS received grants from BMGF. RC received support from EC Horizon for attending the meetings and/or travel, and is a member of the Steering Committees, together with other representatives of the Vacc-iNTS consortium partners, of the EC Horizon 2020 and the EDCTP grants awarded to the iNTS-GMMA vaccine project. BH received a VASE Travel Grant in 2022. MR is a principal investigator across multiple vaccine trials, a member of a DSMB and the JCVI. XL is a member of the DSMB across multiple trials, a clinical trial steering committee, the WHO SAGE Typhoid Working Group, and the WHO Technical Advisory Group for *Salmonella* Vaccines. MR and LG received support from the Horizon 2020 grant to attend meetings. The authors declare no other financial or non-financial relationships or activities.

The Vacc-iNTS consortium was funded by the same grant funding the study. EU Framework Programme for Research and Innovation grant, Horizon 2020 (grant agreement number 815439).

## Appendix A Supplementary data

Vacc-iNTS Consortium Members

Suppl. Figures and Tables
