# Supplementary material for: Safety and immunogenicity of the invasive non-typhoidal Salmonella (iNTS)-GMMA vaccine: a first-in-human, randomised, dose escalation trial
Source: eBioMedicine. 2025 Sep 3;119:105903. doi: 10.1016/j.ebiom.2025.105903 (PMC12444187; doi:10.1016/j.ebiom.2025.105903)
Supplement: Vacc-iNTS Consortium Members [file mmc1.docx]

**Vacc-iNTS Consortium**

| First Names | Surname |
| --- | --- |
| Francis | Agyapong |
| Gianluca | Breghi |
| Annalisa | Ciabattini |
| John A. | Crump |
| Melita A | Gordon |
| Liselotte | Hardy |
| Samuel | Kariuki |
| Stefano | Malvolti |
| Carsten | Mantel |
| Christian S. | Marchello |
| Florian | Marks |
| Donata | Medaglini |
| Tonney S. | Nyirenda |
| Mercy | Ngetich |
| Ellis | Owusu-Dabo |
| Francesco | Santoro |
| J. Anthony G. | Scott |
| Bassiahi | Abdramane Soura |
| Tiziana | Spadafina |
| Bieke | Tack |
