# Supplementary material for: Safety and immunogenicity of the invasive non-typhoidal Salmonella (iNTS)-GMMA vaccine: a first-in-human, randomised, dose escalation trial
Source: eBioMedicine. 2025 Sep 3;119:105903. doi: 10.1016/j.ebiom.2025.105903 (PMC12444187; doi:10.1016/j.ebiom.2025.105903)
Supplement: Suppl. Figures and Tables [file mmc2.pdf]

Supplementary Material 1

Figure 1 Reactogenicity – solicited local adverse reactions following first vaccination by study arm. 2
Figure 2 Reactogenicity – solicited local adverse reactions following second vaccination by study arm. 9
Figure 3 Reactogenicity – solicited local adverse reactions following third vaccination by study arm. 10
Figure 4 Reactogenicity – solicited systemic adverse reactions following first vaccination by study arm. 11
Figure 5 Reactogenicity – solicited systemic adverse reactions following second vaccination by study arm. 12
Figure 6 Reactogenicity – solicited systemic adverse reactions following third vaccination by study arm. 13

Table 1 Summary of participants with solicited systemic and administration site events by maximum intensity within 7 days after each and any vaccination 2
Table 2 Related unsolicited adverse events 14
Table 3 Unsolicited adverse events of grade 3+ severity 15
Table 4 Immune responses – geometric mean concentrations for ELISA by study, timepoint, and strain. 16
Table 5 Immune responses – geometric mean concentrations for serum bactericidal antibody assay by study, timepoint, and strain. 18
Table 6 Seroresponse rate with 95% CI, defined as the percentage of subjects with post-vaccination titers equal to or exceeding 4 times the baseline for serum bactericidal antibody, by study group and timepoint. 19

Table 1 Summary of participants with solicited systemic and administration site events by maximum intensity within 7 days after each and any vaccination

Solicited adverse event	Severity Grade	Dose 1			Dose 2			Dose 3			Any dose		
		Full dose	Low dose	Placebo	Full dose	Low dose	Placebo	Full dose	Low dose	Placebo	Full dose	Low dose	Placebo
Abdominal pain	None	15 (100%) [95% CI: 78% - 100%]	3 (75%) [95% CI: 19% - 99%]	10 (83.3%) [95% CI: 52% - 98%]	14 (93.3%) [95% CI: 68% - 100%]	2 (66.7%) [95% CI: 9% - 99%]	10 (83.3%) [95% CI: 52% - 98%]	9 (90%) [95% CI: 55% - 100%]	1 (33.3%) [95% CI: 1% - 91%]	10 (90.9%) [95% CI: 59% - 100%]	13 (86.7%) [95% CI: 60% - 98%]	2 (50%) [95% CI: 7% - 93%]	9 (75%) [95% CI: 43% - 95%]
Abdominal pain	Mild	NA	1 (25%) [95% CI: 1% - 81%]	2 (16.7%) [95% CI: 2% - 48%]	1 (6.7%) [95% CI: 0% - 32%]	1 (33.3%) [95% CI: 1% - 91%]	2 (16.7%) [95% CI: 2% - 48%]	NA	2 (66.7%) [95% CI: 9% - 99%]	1 (9.1%) [95% CI: 0% - 41%]	1 (6.7%) [95% CI: 0% - 32%]	2 (50%) [95% CI: 7% - 93%]	3 (25%) [95% CI: 5% - 57%]
Abdominal pain	Moderate	NA	NA	NA	NA	NA	NA	1 (10%) [95% CI: 0% - 45%]	NA	NA	1 (6.7%) [95% CI: 0% - 32%]	NA	NA
Abdominal pain	Severe	NA	NA	NA	NA	NA	NA	NA	NA	NA	NA	NA	NA
Chills	None	8 (53.3%) [95% CI: 27% - 79%]	3 (75%) [95% CI: 19% - 99%]	12 (100%) [95% CI: 74% - 100%]	10 (66.7%) [95% CI: 38% - 88%]	3 (100%) [95% CI: 29% - 100%]	12 (100%) [95% CI: 74% - 100%]	9 (90%) [95% CI: 55% - 100%]	3 (100%) [95% CI: 29% - 100%]	11 (100%) [95% CI: 72% - 100%]	7 (46.7%) [95% CI: 21% - 73%]	3 (75%) [95% CI: 19% - 99%]	12 (100%) [95% CI: 74% - 100%]
Chills	Mild	5 (33.3%) [95% CI: 12% - 62%]	1 (25%) [95% CI: 1% - 81%]	NA	4 (26.7%) [95% CI: 8% - 55%]	NA	NA	1 (10%) [95% CI: 0% - 45%]	NA	NA	6 (40%) [95% CI: 16% - 68%]	1 (25%) [95% CI: 1% - 81%]	NA
Chills	Moderate	2 (13.3%) [95% CI: 2% - 40%]	NA	NA	1 (6.7%) [95% CI: 0% - 32%]	NA	NA	NA	NA	NA	2 (13.3%) [95% CI: 2% - 40%]	NA	NA
Chills	Severe	NA	NA	NA	NA	NA	NA	NA	NA	NA	NA	NA	NA
Diarrhoea	None	14 (93.3%) [95% CI: 68% - 100%]	3 (75%) [95% CI: 19% - 99%]	11 (91.7%) [95% CI: 62% - 100%]	14 (93.3%) [95% CI: 68% - 100%]	2 (66.7%) [95% CI: 9% - 99%]	11 (91.7%) [95% CI: 62% - 100%]	8 (80%) [95% CI: 44% - 97%]	1 (33.3%) [95% CI: 1% - 91%]	9 (81.8%) [95% CI: 48% - 98%]	12 (80%) [95% CI: 52% - 96%]	2 (50%) [95% CI: 7% - 93%]	10 (83.3%) [95% CI: 52% - 98%]
Diarrhoea	Mild	1 (6.7%) [95% CI: 0% - 32%]	NA	1 (8.3%) [95% CI: 0% - 38%]	1 (6.7%) [95% CI: 0% - 32%]	1 (33.3%) [95% CI: 1% - 91%]	1 (8.3%) [95% CI: 0% - 38%]	2 (20%) [95% CI: 3% - 56%]	2 (66.7%) [95% CI: 9% - 99%]	2 (18.2%) [95% CI: 2% - 52%]	3 (20%) [95% CI: 4% - 48%]	1 (25%) [95% CI: 1% - 81%]	2 (16.7%) [95% CI: 2% - 48%]
Diarrhoea	Moderate	NA	NA	NA	NA	NA	NA	NA	NA	NA	NA	NA	NA
Diarrhoea	Severe	NA	1 (25%) [95% CI: 1% - 81%]	NA	NA	NA	NA	NA	NA	NA	NA	1 (25%) [95% CI: 1% - 81%]	NA
Fatigue	None	3 (20%) [95% CI: 4% - 48%]	3 (75%) [95% CI: 19% - 99%]	7 (58.3%) [95% CI: 28% - 85%]	3 (20%) [95% CI: 4% - 48%]	3 (100%) [95% CI: 29% - 100%]	11 (91.7%) [95% CI: 62% - 100%]	4 (40%) [95% CI: 12% - 74%]	3 (100%) [95% CI: 29% - 100%]	6 (54.5%) [95% CI: 23% - 83%]	2 (13.3%) [95% CI: 2% - 40%]	3 (75%) [95% CI: 19% - 99%]	5 (41.7%) [95% CI: 15% - 72%]

Solicited adverse event	Severity Grade	Dose 1			Dose 2			Dose 3			Any dose		
		Full dose	Low dose	Placebo	Full dose	Low dose	Placebo	Full dose	Low dose	Placebo	Full dose	Low dose	Placebo
Fatigue	Mild	7 (46.7%) [95% CI: 21% - 73%]	NA	4 (33.3%) [95% CI: 10% - 65%]	9 (60%) [95% CI: 32% - 84%]	NA	1 (8.3%) [95% CI: 0% - 38%]	2 (20%) [95% CI: 3% - 56%]	NA	4 (36.4%) [95% CI: 11% - 69%]	5 (33.3%) [95% CI: 12% - 62%]	NA	5 (41.7%) [95% CI: 15% - 72%]
Fatigue	Moderate	5 (33.3%) [95% CI: 12% - 62%]	1 (25%) [95% CI: 1% - 81%]	1 (8.3%) [95% CI: 0% - 38%]	3 (20%) [95% CI: 4% - 48%]	NA	NA	3 (30%) [95% CI: 7% - 65%]	NA	NA	7 (46.7%) [95% CI: 21% - 73%]	1 (25%) [95% CI: 1% - 81%]	1 (8.3%) [95% CI: 0% - 38%]
Fatigue	Severe	NA	NA	NA	NA	NA	NA	1 (10%) [95% CI: 0% - 45%]	NA	1 (9.1%) [95% CI: 0% - 41%]	1 (6.7%) [95% CI: 0% - 32%]	NA	1 (8.3%) [95% CI: 0% - 38%]
Fever	None	12 (80%) [95% CI: 52% - 96%]	3 (75%) [95% CI: 19% - 99%]	12 (100%) [95% CI: 74% - 100%]	14 (93.3%) [95% CI: 68% - 100%]	3 (100%) [95% CI: 29% - 100%]	12 (100%) [95% CI: 74% - 100%]	9 (90%) [95% CI: 55% - 100%]	3 (100%) [95% CI: 29% - 100%]	11 (100%) [95% CI: 72% - 100%]	11 (73.3%) [95% CI: 45% - 92%]	3 (75%) [95% CI: 19% - 99%]	12 (100%) [95% CI: 74% - 100%]
Fever	Mild	2 (13.3%) [95% CI: 2% - 40%]	1 (25%) [95% CI: 1% - 81%]	NA	1 (6.7%) [95% CI: 0% - 32%]	NA	NA	1 (10%) [95% CI: 0% - 45%]	NA	NA	3 (20%) [95% CI: 4% - 48%]	1 (25%) [95% CI: 1% - 81%]	NA
Fever	Moderate	1 (6.7%) [95% CI: 0% - 32%]	NA	NA	NA	NA	NA	NA	NA	NA	1 (6.7%) [95% CI: 0% - 32%]	NA	NA
Fever	Severe	NA	NA	NA	NA	NA	NA	NA	NA	NA	NA	NA	NA
Feverishness	None	10 (66.7%) [95% CI: 38% - 88%]	3 (75%) [95% CI: 19% - 99%]	12 (100%) [95% CI: 74% - 100%]	11 (73.3%) [95% CI: 45% - 92%]	3 (100%) [95% CI: 29% - 100%]	12 (100%) [95% CI: 74% - 100%]	9 (90%) [95% CI: 55% - 100%]	2 (66.7%) [95% CI: 9% - 99%]	11 (100%) [95% CI: 72% - 100%]	8 (53.3%) [95% CI: 27% - 79%]	3 (75%) [95% CI: 19% - 99%]	12 (100%) [95% CI: 74% - 100%]
Feverishness	Mild	5 (33.3%) [95% CI: 12% - 62%]	1 (25%) [95% CI: 1% - 81%]	NA	3 (20%) [95% CI: 4% - 48%]	NA	NA	1 (10%) [95% CI: 0% - 45%]	NA	NA	6 (40%) [95% CI: 16% - 68%]	NA	NA
Feverishness	Moderate	NA	NA	NA	1 (6.7%) [95% CI: 0% - 32%]	NA	NA	NA	1 (33.3%) [95% CI: 1% - 91%]	NA	1 (6.7%) [95% CI: 0% - 32%]	1 (25%) [95% CI: 1% - 81%]	NA
Feverishness	Severe	NA	NA	NA	NA	NA	NA	NA	NA	NA	NA	NA	NA
Hardness	None	14 (93.3%) [95% CI: 68% - 100%]	4 (100%) [95% CI: 40% - 100%]	12 (100%) [95% CI: 74% - 100%]	14 (93.3%) [95% CI: 68% - 100%]	3 (100%) [95% CI: 29% - 100%]	12 (100%) [95% CI: 74% - 100%]	10 (100%) [95% CI: 69% - 100%]	2 (66.7%) [95% CI: 9% - 99%]	11 (100%) [95% CI: 72% - 100%]	14 (93.3%) [95% CI: 68% - 100%]	3 (75%) [95% CI: 19% - 99%]	12 (100%) [95% CI: 74% - 100%]
Hardness	Mild	1 (6.7%) [95% CI: 0% - 32%]	NA	NA	1 (6.7%) [95% CI: 0% - 32%]	NA	NA	NA	1 (33.3%) [95% CI: 1% - 91%]	NA	1 (6.7%) [95% CI: 0% - 32%]	1 (25%) [95% CI: 1% - 81%]	NA
Hardness	Moderate	NA	NA	NA	NA	NA	NA	NA	NA	NA	NA	NA	NA
Hardness	Severe	NA	NA	NA	NA	NA	NA	NA	NA	NA	NA	NA	NA

Solicited adverse event	Severity Grade	Dose 1			Dose 2			Dose 3			Any dose		
		Full dose	Low dose	Placebo	Full dose	Low dose	Placebo	Full dose	Low dose	Placebo	Full dose	Low dose	Placebo
Headache	None	4 (26.7%) [95% CI: 8% - 55%]	3 (75%) [95% CI: 19% - 99%]	7 (58.3%) [95% CI: 28% - 85%]	10 (66.7%) [95% CI: 38% - 88%]	1 (33.3%) [95% CI: 1% - 91%]	10 (83.3%) [95% CI: 52% - 98%]	5 (50%) [95% CI: 19% - 81%]	1 (33.3%) [95% CI: 1% - 91%]	9 (81.8%) [95% CI: 48% - 98%]	2 (13.3%) [95% CI: 2% - 40%]	2 (50%) [95% CI: 7% - 93%]	5 (41.7%) [95% CI: 15% - 72%]
Headache	Mild	9 (60%) [95% CI: 32% - 84%]	NA	4 (33.3%) [95% CI: 10% - 65%]	NA	1 (33.3%) [95% CI: 1% - 91%]	2 (16.7%) [95% CI: 2% - 48%]	3 (30%) [95% CI: 7% - 65%]	1 (33.3%) [95% CI: 1% - 91%]	NA	6 (40%) [95% CI: 16% - 68%]	1 (25%) [95% CI: 1% - 81%]	4 (33.3%) [95% CI: 10% - 65%]
Headache	Moderate	2 (13.3%) [95% CI: 2% - 40%]	1 (25%) [95% CI: 1% - 81%]	1 (8.3%) [95% CI: 0% - 38%]	4 (26.7%) [95% CI: 8% - 55%]	1 (33.3%) [95% CI: 1% - 91%]	NA	2 (20%) [95% CI: 3% - 56%]	NA	2 (18.2%) [95% CI: 2% - 52%]	6 (40%) [95% CI: 16% - 68%]	NA	3 (25%) [95% CI: 5% - 57%]
Headache	Severe	NA	NA	NA	1 (6.7%) [95% CI: 0% - 32%]	NA	NA	NA	1 (33.3%) [95% CI: 1% - 91%]	NA	1 (6.7%) [95% CI: 0% - 32%]	1 (25%) [95% CI: 1% - 81%]	NA
Joint pain	None	13 (86.7%) [95% CI: 60% - 98%]	3 (75%) [95% CI: 19% - 99%]	11 (91.7%) [95% CI: 62% - 100%]	11 (73.3%) [95% CI: 45% - 92%]	3 (100%) [95% CI: 29% - 100%]	12 (100%) [95% CI: 74% - 100%]	8 (80%) [95% CI: 44% - 97%]	3 (100%) [95% CI: 29% - 100%]	11 (100%) [95% CI: 72% - 100%]	11 (73.3%) [95% CI: 45% - 92%]	3 (75%) [95% CI: 19% - 99%]	11 (91.7%) [95% CI: 62% - 100%]
Joint pain	Mild	2 (13.3%) [95% CI: 2% - 40%]	1 (25%) [95% CI: 1% - 81%]	1 (8.3%) [95% CI: 0% - 38%]	2 (13.3%) [95% CI: 2% - 40%]	NA	NA	NA	NA	NA	1 (6.7%) [95% CI: 0% - 32%]	1 (25%) [95% CI: 1% - 81%]	1 (8.3%) [95% CI: 0% - 38%]
Joint pain	Moderate	NA	NA	NA	2 (13.3%) [95% CI: 2% - 40%]	NA	NA	2 (20%) [95% CI: 3% - 56%]	NA	NA	3 (20%) [95% CI: 4% - 48%]	NA	NA
Joint pain	Severe	NA	NA	NA	NA	NA	NA	NA	NA	NA	NA	NA	NA
Loss of appetite	None	13 (86.7%) [95% CI: 60% - 98%]	3 (75%) [95% CI: 19% - 99%]	11 (91.7%) [95% CI: 62% - 100%]	11 (73.3%) [95% CI: 45% - 92%]	2 (66.7%) [95% CI: 9% - 99%]	12 (100%) [95% CI: 74% - 100%]	10 (100%) [95% CI: 69% - 100%]	2 (66.7%) [95% CI: 9% - 99%]	10 (90.9%) [95% CI: 59% - 100%]	9 (60%) [95% CI: 32% - 84%]	3 (75%) [95% CI: 19% - 99%]	10 (83.3%) [95% CI: 52% - 98%]
Loss of appetite	Mild	2 (13.3%) [95% CI: 2% - 40%]	NA	1 (8.3%) [95% CI: 0% - 38%]	4 (26.7%) [95% CI: 8% - 55%]	1 (33.3%) [95% CI: 1% - 91%]	NA	NA	1 (33.3%) [95% CI: 1% - 91%]	1 (9.1%) [95% CI: 0% - 41%]	6 (40%) [95% CI: 16% - 68%]	NA	2 (16.7%) [95% CI: 2% - 48%]
Loss of appetite	Moderate	NA	1 (25%) [95% CI: 1% - 81%]	NA	NA	NA	NA	NA	NA	NA	NA	1 (25%) [95% CI: 1% - 81%]	NA
Loss of appetite	Severe	NA	NA	NA	NA	NA	NA	NA	NA	NA	NA	NA	NA
Malaise	None	4 (26.7%) [95% CI: 8% - 55%]	3 (75%) [95% CI: 19% - 99%]	10 (83.3%) [95% CI: 52% - 98%]	6 (40%) [95% CI: 16% - 68%]	3 (100%) [95% CI: 29% - 100%]	12 (100%) [95% CI: 74% - 100%]	7 (70%) [95% CI: 35% - 93%]	3 (100%) [95% CI: 29% - 100%]	9 (81.8%) [95% CI: 48% - 98%]	3 (20%) [95% CI: 4% - 48%]	3 (75%) [95% CI: 19% - 99%]	10 (83.3%) [95% CI: 52% - 98%]
Malaise	Mild	7 (46.7%) [95% CI: 21% - 73%]	NA	2 (16.7%) [95% CI: 2% - 48%]	6 (40%) [95% CI: 16% - 68%]	NA	NA	1 (10%) [95% CI: 0% - 45%]	NA	NA	7 (46.7%) [95% CI: 21% - 73%]	NA	NA

Solicited adverse event	Severity Grade	Dose 1			Dose 2			Dose 3			Any dose		
		Full dose	Low dose	Placebo	Full dose	Low dose	Placebo	Full dose	Low dose	Placebo	Full dose	Low dose	Placebo
Malaise	Moderate	3 (20%) [95% CI: 4% - 48%]	1 (25%) [95% CI: 1% - 81%]	NA	1 (6.7%) [95% CI: 0% - 32%]	NA	NA	2 (20%) [95% CI: 3% - 56%]	NA	1 (9.1%) [95% CI: 0% - 41%]	2 (13.3%) [95% CI: 2% - 40%]	1 (25%) [95% CI: 1% - 81%]	1 (8.3%) [95% CI: 0% - 38%]
Malaise	Severe	1 (6.7%) [95% CI: 0% - 32%]	NA	NA	2 (13.3%) [95% CI: 2% - 40%]	NA	NA	NA	NA	1 (9.1%) [95% CI: 0% - 41%]	3 (20%) [95% CI: 4% - 48%]	NA	1 (8.3%) [95% CI: 0% - 38%]
Muscle aches	None	5 (33.3%) [95% CI: 12% - 62%]	2 (50%) [95% CI: 7% - 93%]	9 (75%) [95% CI: 43% - 95%]	5 (33.3%) [95% CI: 12% - 62%]	2 (66.7%) [95% CI: 9% - 99%]	11 (91.7%) [95% CI: 62% - 100%]	5 (50%) [95% CI: 19% - 81%]	2 (66.7%) [95% CI: 9% - 99%]	11 (100%) [95% CI: 72% - 100%]	2 (13.3%) [95% CI: 2% - 40%]	2 (50%) [95% CI: 7% - 93%]	8 (66.7%) [95% CI: 35% - 90%]
Muscle aches	Mild	5 (33.3%) [95% CI: 12% - 62%]	2 (50%) [95% CI: 7% - 93%]	2 (16.7%) [95% CI: 2% - 48%]	7 (46.7%) [95% CI: 21% - 73%]	1 (33.3%) [95% CI: 1% - 91%]	1 (8.3%) [95% CI: 0% - 38%]	3 (30%) [95% CI: 7% - 65%]	NA	NA	7 (46.7%) [95% CI: 21% - 73%]	1 (25%) [95% CI: 1% - 81%]	3 (25%) [95% CI: 5% - 57%]
Muscle aches	Moderate	5 (33.3%) [95% CI: 12% - 62%]	NA	NA	3 (20%) [95% CI: 4% - 48%]	NA	NA	2 (20%) [95% CI: 3% - 56%]	1 (33.3%) [95% CI: 1% - 91%]	NA	6 (40%) [95% CI: 16% - 68%]	1 (25%) [95% CI: 1% - 81%]	NA
Muscle aches	Severe	NA	NA	1 (8.3%) [95% CI: 0% - 38%]	NA	NA	NA	NA	NA	NA	NA	NA	1 (8.3%) [95% CI: 0% - 38%]
Nausea	None	14 (93.3%) [95% CI: 68% - 100%]	3 (75%) [95% CI: 19% - 99%]	8 (66.7%) [95% CI: 35% - 90%]	13 (86.7%) [95% CI: 60% - 98%]	2 (66.7%) [95% CI: 9% - 99%]	12 (100%) [95% CI: 74% - 100%]	8 (80%) [95% CI: 44% - 97%]	2 (66.7%) [95% CI: 9% - 99%]	9 (81.8%) [95% CI: 48% - 98%]	11 (73.3%) [95% CI: 45% - 92%]	3 (75%) [95% CI: 19% - 99%]	7 (58.3%) [95% CI: 28% - 85%]
Nausea	Mild	1 (6.7%) [95% CI: 0% - 32%]	NA	4 (33.3%) [95% CI: 10% - 65%]	1 (6.7%) [95% CI: 0% - 32%]	1 (33.3%) [95% CI: 1% - 91%]	NA	1 (10%) [95% CI: 0% - 45%]	NA	2 (18.2%) [95% CI: 2% - 52%]	3 (20%) [95% CI: 4% - 48%]	NA	5 (41.7%) [95% CI: 15% - 72%]
Nausea	Moderate	NA	1 (25%) [95% CI: 1% - 81%]	NA	1 (6.7%) [95% CI: 0% - 32%]	NA	NA	NA	1 (33.3%) [95% CI: 1% - 91%]	NA	NA	1 (25%) [95% CI: 1% - 81%]	NA
Nausea	Severe	NA	NA	NA	NA	NA	NA	1 (10%) [95% CI: 0% - 45%]	NA	NA	1 (6.7%) [95% CI: 0% - 32%]	NA	NA
Pain	None	2 (13.3%) [95% CI: 2% - 40%]	2 (50%) [95% CI: 7% - 93%]	8 (66.7%) [95% CI: 35% - 90%]	1 (6.7%) [95% CI: 0% - 32%]	1 (33.3%) [95% CI: 1% - 91%]	12 (100%) [95% CI: 74% - 100%]	1 (10%) [95% CI: 0% - 45%]	NA	9 (81.8%) [95% CI: 48% - 98%]	1 (6.7%) [95% CI: 0% - 32%]	1 (25%) [95% CI: 1% - 81%]	6 (50%) [95% CI: 21% - 79%]
Pain	Mild	4 (26.7%) [95% CI: 8% - 55%]	1 (25%) [95% CI: 1% - 81%]	4 (33.3%) [95% CI: 10% - 65%]	12 (80%) [95% CI: 52% - 96%]	2 (66.7%) [95% CI: 9% - 99%]	NA	6 (60%) [95% CI: 26% - 88%]	2 (66.7%) [95% CI: 9% - 99%]	1 (9.1%) [95% CI: 0% - 41%]	4 (26.7%) [95% CI: 8% - 55%]	2 (50%) [95% CI: 7% - 93%]	5 (41.7%) [95% CI: 15% - 72%]
Pain	Moderate	9 (60%) [95% CI: 32% - 84%]	1 (25%) [95% CI: 1% - 81%]	NA	2 (13.3%) [95% CI: 2% - 40%]	NA	NA	3 (30%) [95% CI: 7% - 65%]	1 (33.3%) [95% CI: 1% - 91%]	1 (9.1%) [95% CI: 0% - 41%]	10 (66.7%) [95% CI: 38% - 88%]	1 (25%) [95% CI: 1% - 81%]	1 (8.3%) [95% CI: 0% - 38%]
Pain	Severe	NA	NA	NA	NA	NA	NA	NA	NA	NA	NA	NA	NA

Solicited adverse event	Severity Grade	Dose 1			Dose 2			Dose 3			Any dose		
		Full dose	Low dose	Placebo	Full dose	Low dose	Placebo	Full dose	Low dose	Placebo	Full dose	Low dose	Placebo
Rash	None	12 (80%) [95% CI: 52% - 96%]	4 (100%) [95% CI: 40% - 100%]	12 (100%) [95% CI: 74% - 100%]	11 (73.3%) [95% CI: 45% - 92%]	2 (66.7%) [95% CI: 9% - 99%]	12 (100%) [95% CI: 74% - 100%]	10 (100%) [95% CI: 69% - 100%]	3 (100%) [95% CI: 29% - 100%]	11 (100%) [95% CI: 72% - 100%]	9 (60%) [95% CI: 32% - 84%]	3 (75%) [95% CI: 19% - 99%]	12 (100%) [95% CI: 74% - 100%]
Rash	Mild	2 (13.3%) [95% CI: 2% - 40%]	NA	NA	3 (20%) [95% CI: 4% - 48%]	1 (33.3%) [95% CI: 1% - 91%]	NA	NA	NA	NA	5 (33.3%) [95% CI: 12% - 62%]	1 (25%) [95% CI: 1% - 81%]	NA
Rash	Moderate	1 (6.7%) [95% CI: 0% - 32%]	NA	NA	1 (6.7%) [95% CI: 0% - 32%]	NA	NA	NA	NA	NA	1 (6.7%) [95% CI: 0% - 32%]	NA	NA
Rash	Severe	NA	NA	NA	NA	NA	NA	NA	NA	NA	NA	NA	NA
Redness	None	11 (73.3%) [95% CI: 45% - 92%]	4 (100%) [95% CI: 40% - 100%]	12 (100%) [95% CI: 74% - 100%]	15 (100%) [95% CI: 78% - 100%]	2 (66.7%) [95% CI: 9% - 99%]	12 (100%) [95% CI: 74% - 100%]	10 (100%) [95% CI: 69% - 100%]	3 (100%) [95% CI: 29% - 100%]	11 (100%) [95% CI: 72% - 100%]	11 (73.3%) [95% CI: 45% - 92%]	3 (75%) [95% CI: 19% - 99%]	12 (100%) [95% CI: 74% - 100%]
Redness	Mild	1 (6.7%) [95% CI: 0% - 32%]	NA	NA	NA	NA	NA	NA	NA	NA	1 (6.7%) [95% CI: 0% - 32%]	NA	NA
Redness	Moderate	NA	NA	NA	NA	1 (33.3%) [95% CI: 1% - 91%]	NA	NA	NA	NA	NA	1 (25%) [95% CI: 1% - 81%]	NA
Redness	Severe	3 (20%) [95% CI: 4% - 48%]	NA	NA	NA	NA	NA	NA	NA	NA	3 (20%) [95% CI: 4% - 48%]	NA	NA
Swelling	None	13 (86.7%) [95% CI: 60% - 98%]	4 (100%) [95% CI: 40% - 100%]	12 (100%) [95% CI: 74% - 100%]	14 (93.3%) [95% CI: 68% - 100%]	2 (66.7%) [95% CI: 9% - 99%]	12 (100%) [95% CI: 74% - 100%]	9 (90%) [95% CI: 55% - 100%]	3 (100%) [95% CI: 29% - 100%]	11 (100%) [95% CI: 72% - 100%]	13 (86.7%) [95% CI: 60% - 98%]	3 (75%) [95% CI: 19% - 99%]	12 (100%) [95% CI: 74% - 100%]
Swelling	Mild	1 (6.7%) [95% CI: 0% - 32%]	NA	NA	1 (6.7%) [95% CI: 0% - 32%]	NA	NA	1 (10%) [95% CI: 0% - 45%]	NA	NA	1 (6.7%) [95% CI: 0% - 32%]	NA	NA
Swelling	Moderate	NA	NA	NA	NA	1 (33.3%) [95% CI: 1% - 91%]	NA	NA	NA	NA	NA	1 (25%) [95% CI: 1% - 81%]	NA
Swelling	Severe	1 (6.7%) [95% CI: 0% - 32%]	NA	NA	NA	NA	NA	NA	NA	NA	1 (6.7%) [95% CI: 0% - 32%]	NA	NA
Tenderness	None	NA	NA	10 (83.3%) [95% CI: 52% - 98%]	1 (6.7%) [95% CI: 0% - 32%]	NA	10 (83.3%) [95% CI: 52% - 98%]	NA	NA	10 (90.9%) [95% CI: 59% - 100%]	NA	NA	9 (75%) [95% CI: 43% - 95%]
Tenderness	Mild	7 (46.7%) [95% CI: 21% - 73%]	2 (50%) [95% CI: 7% - 93%]	2 (16.7%) [95% CI: 2% - 48%]	7 (46.7%) [95% CI: 21% - 73%]	3 (100%) [95% CI: 29% - 100%]	2 (16.7%) [95% CI: 2% - 48%]	7 (70%) [95% CI: 35% - 93%]	2 (66.7%) [95% CI: 9% - 99%]	1 (9.1%) [95% CI: 0% - 41%]	5 (33.3%) [95% CI: 12% - 62%]	2 (50%) [95% CI: 7% - 93%]	3 (25%) [95% CI: 5% - 57%]

Solicited adverse event	Severity Grade	Dose 1			Dose 2			Dose 3			Any dose		
		**Full dose**	**Low dose**	**Placebo**	**Full dose**	**Low dose**	**Placebo**	**Full dose**	**Low dose**	**Placebo**	**Full dose**	**Low dose**	**Placebo**
Tenderness	Moderate	8 (53.3%) [95% CI: 27% - 79%]	2 (50%) [95% CI: 7% - 93%]	NA	7 (46.7%) [95% CI: 21% - 73%]	NA	NA	3 (30%) [95% CI: 7% - 65%]	1 (33.3%) [95% CI: 1% - 91%]	NA	10 (66.7%) [95% CI: 38% - 88%]	2 (50%) [95% CI: 7% - 93%]	NA
Tenderness	Severe	NA	NA	NA	NA	NA	NA	NA	NA	NA	NA	NA	NA
Vomiting	None	15 (100%) [95% CI: 78% - 100%]	4 (100%) [95% CI: 40% - 100%]	12 (100%) [95% CI: 74% - 100%]	14 (93.3%) [95% CI: 68% - 100%]	3 (100%) [95% CI: 29% - 100%]	12 (100%) [95% CI: 74% - 100%]	9 (90%) [95% CI: 55% - 100%]	3 (100%) [95% CI: 29% - 100%]	11 (100%) [95% CI: 72% - 100%]	13 (86.7%) [95% CI: 60% - 98%]	4 (100%) [95% CI: 40% - 100%]	12 (100%) [95% CI: 74% - 100%]
Vomiting	Mild	NA	NA	NA	1 (6.7%) [95% CI: 0% - 32%]	NA	NA	1 (10%) [95% CI: 0% - 45%]	NA	NA	2 (13.3%) [95% CI: 2% - 40%]	NA	NA
Vomiting	Moderate	NA	NA	NA	NA	NA	NA	NA	NA	NA	NA	NA	NA
Vomiting	Severe	NA	NA	NA	NA	NA	NA	NA	NA	NA	NA	NA	NA

Figure 1 Reactogenicity – solicited local adverse reactions following first vaccination by study arm.

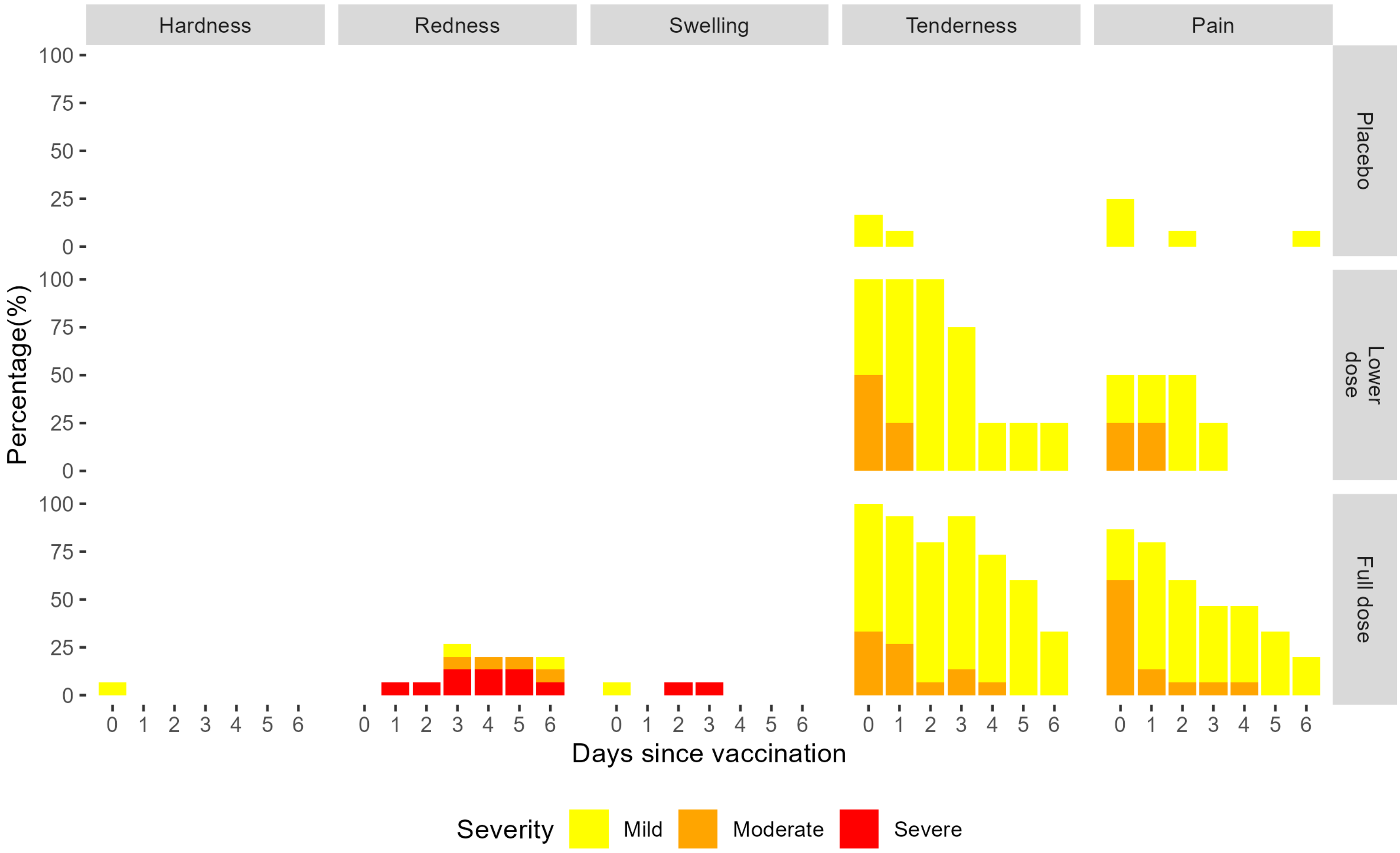

Redness 2.5 – 5 cm; 5.1 – 10 cm; > 10 cm Necrosis or exfoliative dermatitis
Hardness/Swelling 2.5 – 5 cm and does not interfere with activity; 5.1 – 10 cm or interferes with activity; > 10 cm or prevents daily activity

Figure 2 Reactogenicity – solicited local adverse reactions following second vaccination by study arm.

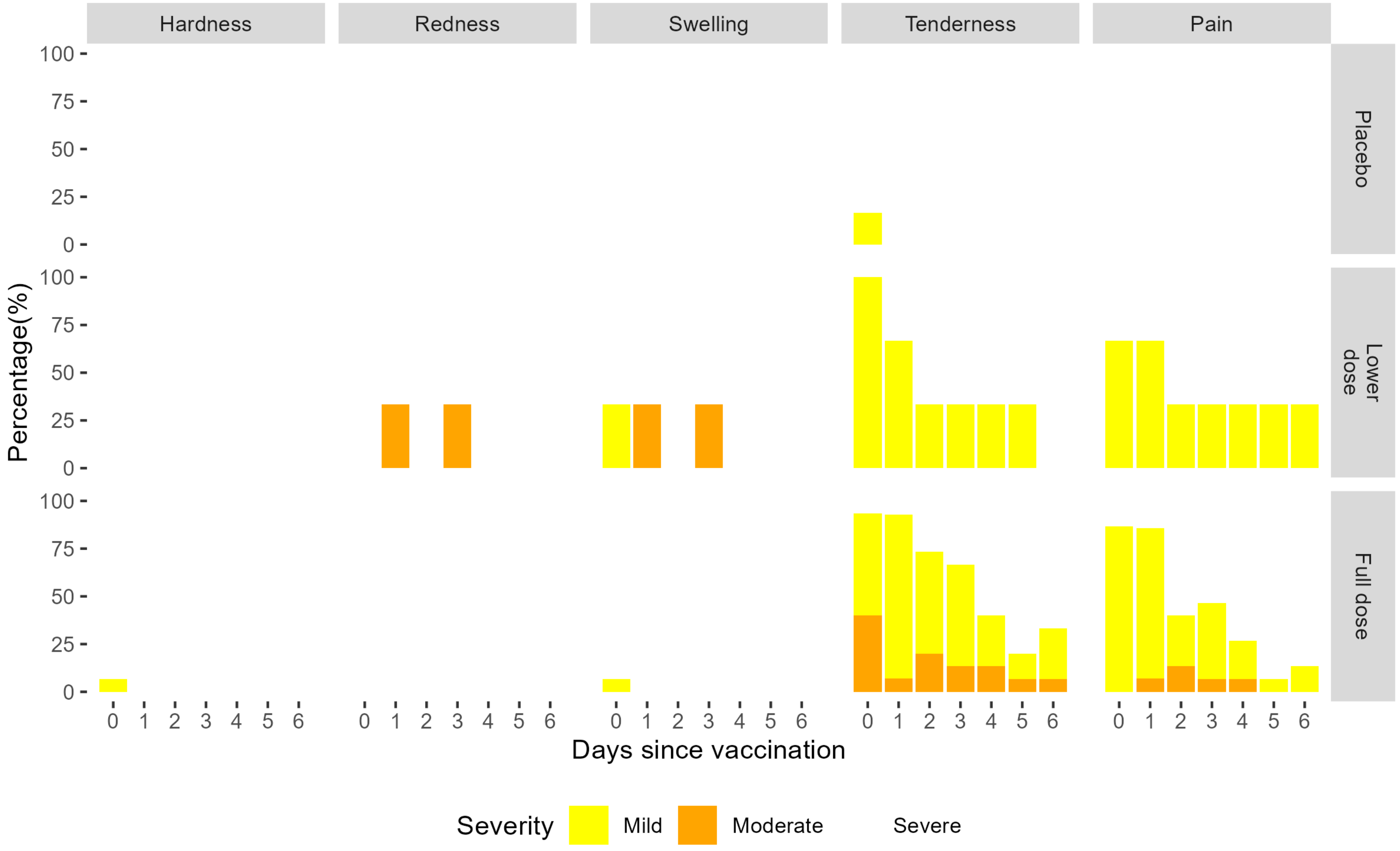

Figure 3 Reactogenicity – solicited local adverse reactions following third vaccination by study arm.

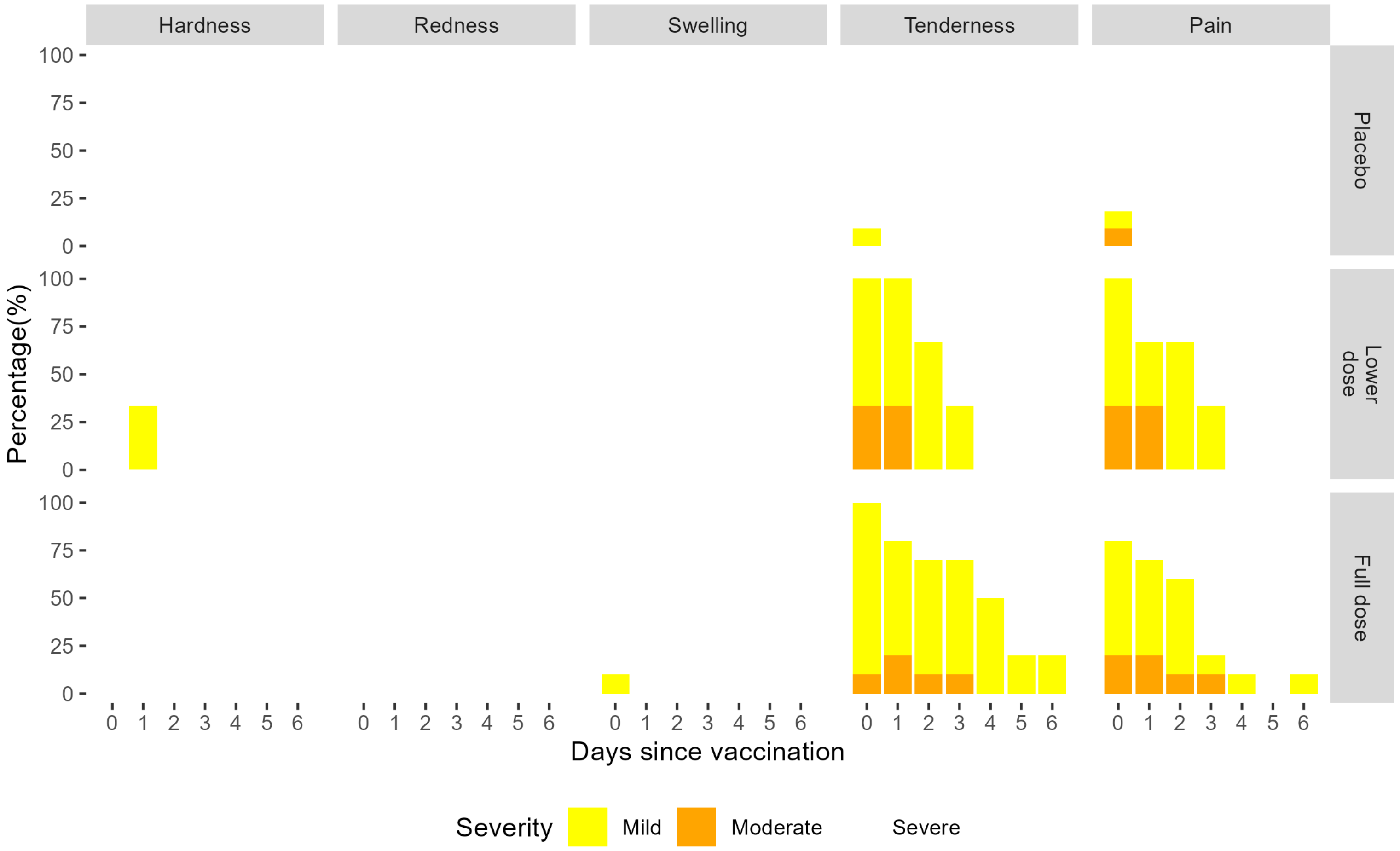

Figure 4 Reactogenicity – solicited systemic adverse reactions following first vaccination by study arm.

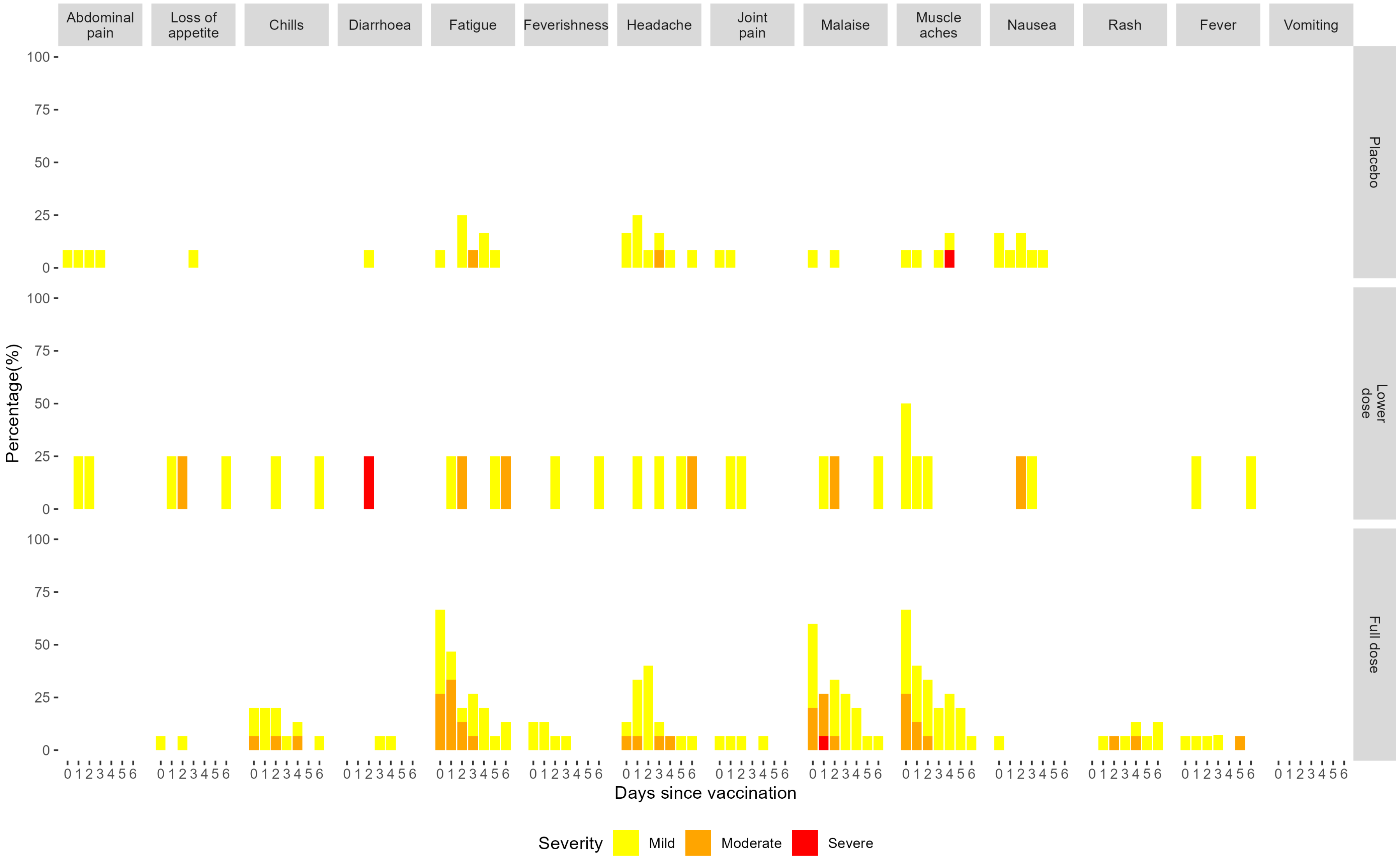

Figure 5 Reactogenicity – solicited systemic adverse reactions following second vaccination by study arm.

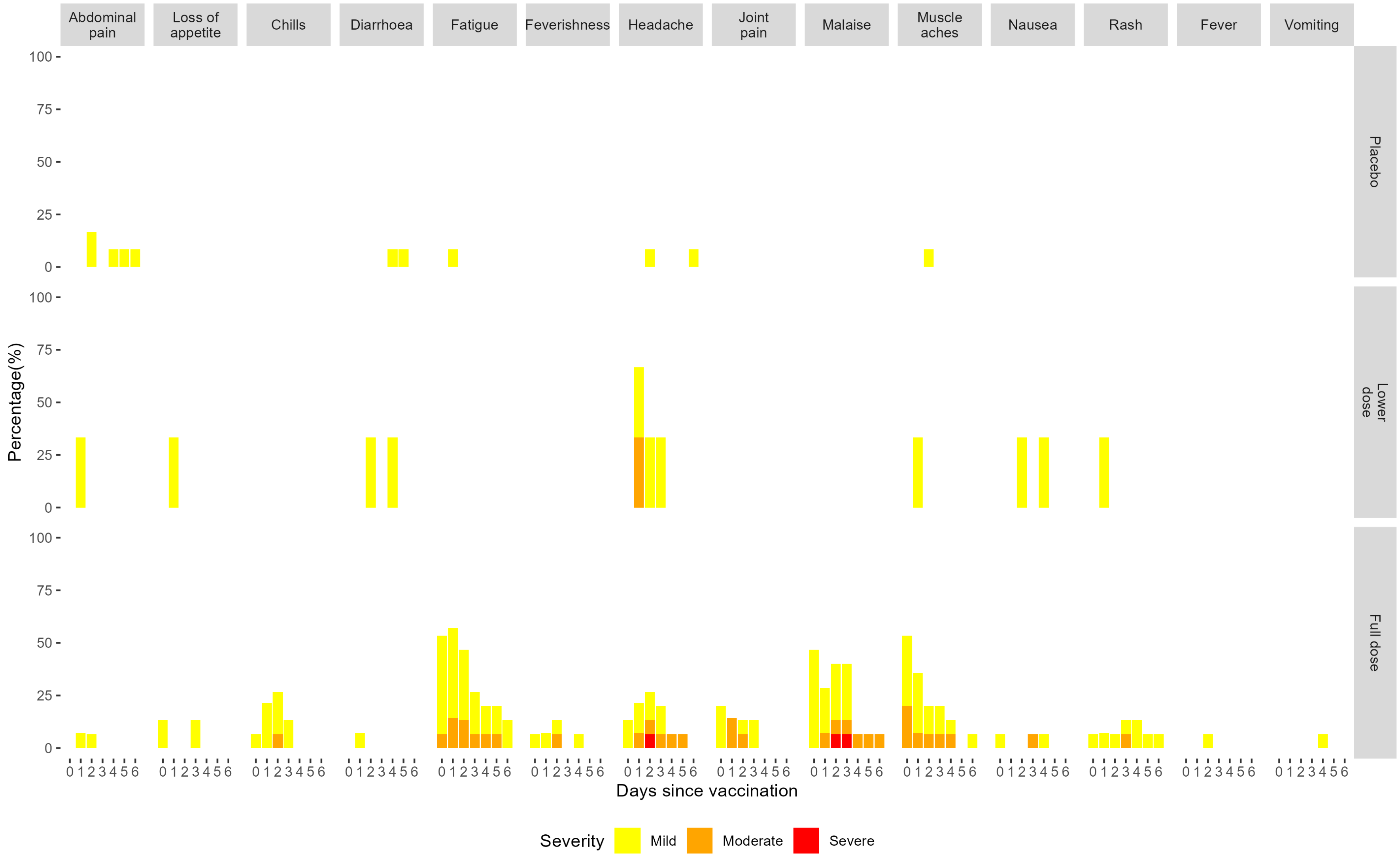

Figure 6 Reactogenicity – solicited systemic adverse reactions following third vaccination by study arm.

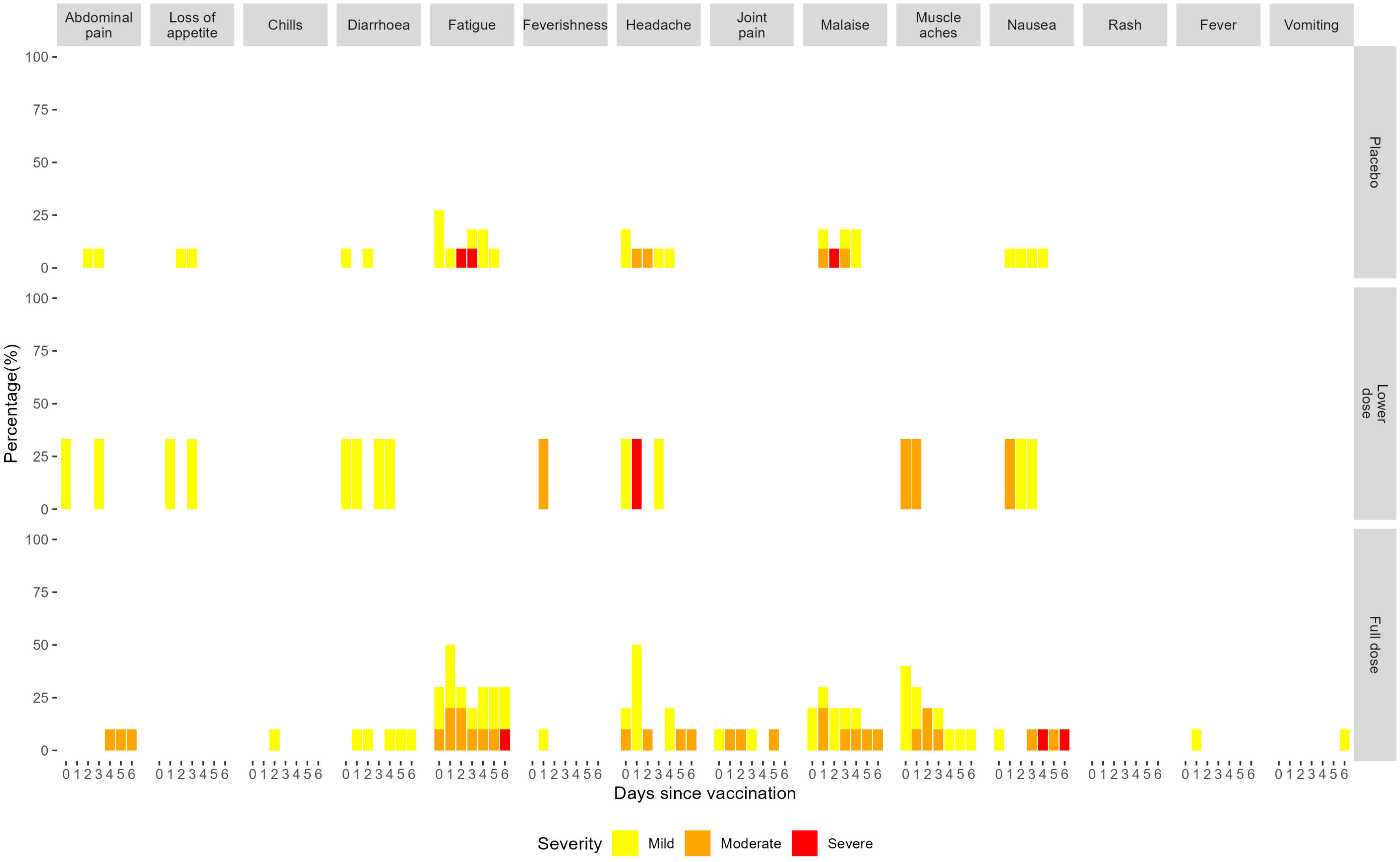

Table 2 Related unsolicited adverse events

Number	Study arm	Diary [illegible]	Which [illegible]	Diagnosis	Severity	Causality	Duration of AE
1	Placebo	No	NA	Neutropenia	Mild	Possible	169
2	Placebo	Yes	First	General Weakness	Moderate	Possible	1
3	Lower dose	Yes	First	Diarrhoea	Severe	Possible	1
4	Full dose	No	NA	Neutropenia	Mild	Possible	138
5	Full dose	Yes	Second	Local pruritis	Mild	Possible	Lost to follow up
6	Placebo	Yes	Third	Fatigue	Mild	Definite	1
7	Full dose	No	NA	Muscle discomfort	Mild	Probable	Lost to follow up
8	Full dose	No	NA	Hyperergic localized reaction	Moderate	Definite	10
9	Full dose	No	NA	Neutropenia	Moderate	Possible	Ongoing at end of study
10	Full dose	Yes	First	Injection site reaction	Moderate	Definite	15
11	Full dose	Yes	Second	Injection site reaction	Severe	Definite	28
12	Placebo	No	NA	Transaminitis	Mild	Possible	7

Table 3 Unsolicited adverse events of grade 3+ severity

Number	Study arm	Diary AE	Which diary	Diagnosis	Severity	Causality	Duration of AE
1	Placebo	No	NA	Musculoskeletal back pain	Severe	No relationship	4
2	Lower dose	Yes	First	Diarrhoea	Severe	Possible	1
3	Full dose	No	NA	Lower respiratory tract infection	Severe	No relationship	6
4	Placebo	No	NA	Helicobacter pylori infection	Severe	No relationship	Ongoing at end of study
5	Full dose	Yes	Second	Concussion	Severe	No relationship	60
6	Full dose	Yes	Second	Injection site reaction	Severe	Definite	28

AE: adverse event.

Table 4 Immune responses – geometric mean concentrations (+/- 95% Confidence Interval) for ELISA by study, timepoint, and strain.

Timepoint	Strain	Full dose	Lower dose	Placebo
0 (first vaccination)	SEn	32.9 (13.5, 80.4) [n=15]	18.9 (2.1, 171.7) [n=4]	73.4 (22.9, 234.9) [n=12]
7	SEn	146.3 (42.4, 505) [n=14]	91.5 (0.2, 40632.9) [n=3]	70 (21.9, 224.4) [n=12]
28	SEn	865.4 (404.9, 1849.6) [n=15]	686 (251.2, 1873.2) [n=4]	73.7 (22.4, 242.3) [n=12]
56 (second vaccination)	SEn	888 (431.6, 1826.9) [n=15]	746.1 (115.7, 4812.5) [n=3]	75.9 (23.3, 247.3) [n=12]
63	SEn	899.4 (387.5, 2087.4) [n=13]	702.4 (92.5, 5336.5) [n=3]	65.1 (19, 222.6) [n=11]
84	SEn	942.1 (476, 1864.9) [n=15]	715.8 (85.1, 6021) [n=3]	67.4 (19.6, 230.9) [n=12]
168 (third vaccination)	SEn	712 (291.4, 1739.5) [n=12]	545.6 (36.7, 8118.7) [n=3]	79.4 (27.5, 229.1) [n=12]
175	SEn	582.6 (170.4, 1991.4) [n=9]	915.9 (4.9, 170582.6) [n=2]	81.3 (22, 299.8) [n=10]
196	SEn	759.3 (327.5, 1760.4) [n=12]	530.7 (90.1, 3126.9) [n=3]	69.1 (20.3, 235.2) [n=11]
350	SEn	550.5 (200, 1515.3) [n=11]	360.2 (19.1, 6792) [n=3]	66.9 (19.4, 230.9) [n=11]
0 (first vaccination)	STm	24.4 (11, 54) [n=15]	46.7 (4.4, 497.3) [n=4]	40.6 (17.6, 93.7) [n=12]
7	STm	186.7 (64.2, 542.8) [n=14]	100.7 (0.2, 48132.3) [n=3]	40 (17.6, 90.8) [n=12]
28	STm	833.2 (401.8, 1727.9) [n=15]	1734.3 (283.2, 10623.1) [n=4]	41.1 (17.6, 95.5) [n=12]
56 (second vaccination)	STm	811.3 (394.4, 1668.7) [n=15]	2016.3 (73.2, 55571) [n=3]	41.7 (17.9, 97.2) [n=12]
63	STm	921.4 (429.9, 1974.6) [n=13]	1963.4 (66.3, 58180.2) [n=3]	45.4 (18.2, 113.4) [n=11]
84	STm	773.5 (394.1, 1518.1) [n=15]	1858 (89.4, 38599.9) [n=3]	38.6 (16.1, 92.8) [n=12]
168 (third vaccination)	STm	458.7 (182.8, 1151.4) [n=12]	1407.9 (92.6, 21397) [n=3]	29.7 (14.2, 62.3) [n=12]
175	STm	592.4 (232.7, 1508.6) [n=9]	722.2 (60.3, 8646) [n=2]	31.2 (12.4, 78.7) [n=10]
196	STm	457.5 (190, 1101.6) [n=12]	1073.1 (32.8, 35109.6) [n=3]	31.5 (13.3, 74.5) [n=11]
350	STm	459.6 (212, 996.4) [n=11]	620.1 (27.7, 13899.7) [n=3]	29.7 (13, 68.1) [n=11]

*SEn: O:9 IgG ELISA; STm: O:4,5 IgG ELISA

Table 5 Immune responses – geometric mean concentrations for serum bactericidal antibody assay by study, timepoint, and strain.

Timepoint	Strain	Full dose	Lower dose	Placebo
0 (first vaccination)	SEn	9001.8 (4181.6, 19378.2) [n=15]	9001.7 (2244.3, 36104.8) [n=4]	5922.7 (3068.9, 11430.1) [n=12]
28	SEn	38722.7 (14209, 105528.1) [n=15]	86436.3 (9214.8, 810783.2) [n=4]	9976 (4261.1, 23355.5) [n=12]
84	SEn	52435.5 (30816.7, 89220.4) [n=15]	34508.6 (1419.7, 838781.7) [n=3]	9422.5 (5007.1, 17731.3) [n=12]
168 (third vaccination)	SEn	28375.9 (10530.9, 76460.1) [n=12]	28093.6 (2622.3, 300982.3) [n=3]	8196.7 (3750.6, 17913.7) [n=12]
196	SEn	57245.7 (27100.6, 120922.5) [n=12]	49986.7 (17641.9, 141632.8) [n=3]	10504.7 (5598, 19712.1) [n=11]
350	SEn	23000.8 (12625.5, 41902.3) [n=11]	107338 (34539.4, 333574.4) [n=3]	13583.9 (5809.1, 31764.4) [n=11]
0 (first vaccination)	STm	6073.5 (3247.8, 11357.7) [n=15]	2812.9 (279.8, 28276.9) [n=4]	8339.5 (3442.3, 20203.8) [n=12]
28	STm	29989 (18528.6, 48537.9) [n=15]	69153.2 (1555.5, 3074275.3) [n=4]	6694.3 (2742, 16343.6) [n=12]
84	STm	27758.5 (18224.2, 42280.8) [n=15]	40021.7 (8370.3, 191359.5) [n=3]	6215.6 (2798.1, 13807.2) [n=12]
168 (third vaccination)	STm	10758.9 (7057.6, 16401.4) [n=12]	12069.8 (2069.2, 70404.6) [n=3]	2814.9 (1594.2, 4970.2) [n=12]
196	STm	18598.3 (12368.6, 27965.8) [n=12]	11478.9 (2310.8, 57022.7) [n=3]	3979.5 (1712.7, 9246.4) [n=11]
350	STm	25333.7 (16403.3, 39126.1) [n=11]	24967.5 (1997.2, 312123.2) [n=3]	10712.1 (5325, 21548.8) [n=11]

Table 6 Seroresponse rate with 95% CI, defined as the percentage of subjects with post-vaccination titers equal to or exceeding 4 times the baseline for serum bactericidal antibody, by study group and timepoint.

In the following table, D0 presents number (percentage) of participants with a baseline value equal to or greater than LLOQ.

	SEn			STm		
	**Full dose**, N = 15 (**95% CI**)[1]	**Lower dose**, N = 4 (**95% CI**)[1]	**Placebo**, N = 12 (**95% CI**)[1]	**Full dose**, N = 15 (**95% CI**)[1]	**Lower dose**, N = 4 (**95% CI**)[1]	**Placebo**, N = 12 (**95% CI**)[1]
**D0***	15 / 15 (100%)	4 / 4 (100%)	12 / 12 (100%)	15 / 15 (100%)	4 / 4 (100%)	12 / 12 (100%)
**D28**	6 / 15 (40%) (16%, 68%)	3 / 4 (75%) (19%, 99%)	2 / 12 (17%) (2.1%, 48%)	9 / 15 (60%) (32%, 84%)	2 / 4 (50%) (6.8%, 93%)	0 / 12 (0%) (0.00%, 26%)
**D84**	9 / 15 (60%) (32%, 84%)	1 / 3 (33%) (0.84%, 91%)	2 / 12 (17%) (2.1%, 48%)	7 / 15 (47%) (21%, 73%)	2 / 3 (67%) (9.4%, 99%)	0 / 12 (0%) (0.00%, 26%)
**D168 (third vaccination)**	5 / 12 (42%) (15%, 72%)	1 / 3 (33%) (0.84%, 91%)	2 / 12 (17%) (2.1%, 48%)	3 / 12 (25%) (5.5%, 57%)	1 / 3 (33%) (0.84%, 91%)	0 / 12 (0%) (0.00%, 26%)
**D196**	7 / 12 (58%) (28%, 85%)	2 / 3 (67%) (9.4%, 99%)	3 / 11 (27%) (6.0%, 61%)	5 / 12 (42%) (15%, 72%)	1 / 3 (33%) (0.84%, 91%)	0 / 11 (0%) (0.00%, 28%)
**D350**	4 / 11 (36%) (11%, 69%)	2 / 3 (67%) (9.4%, 99%)	5 / 11 (45%) (17%, 77%)	6 / 11 (55%) (23%, 83%)	1 / 3 (33%) (0.84%, 91%)	0 / 11 (0%) (0.00%, 28%)

[1]CI = Exact Confidence Interval

* The number (percentage) of participants with a baseline value equal to or greater than LLOQ.
